# Human Digital Twin for Personalized Elderly Type 2 Diabetes Management

**DOI:** 10.3390/jcm12062094

**Published:** 2023-03-07

**Authors:** Padmapritha Thamotharan, Seshadhri Srinivasan, Jothydev Kesavadev, Gopika Krishnan, Viswanathan Mohan, Subathra Seshadhri, Korkut Bekiroglu, Chiara Toffanin

**Affiliations:** 1 Kalasalingam Academy of Research and Education, Srivilliputhur 626126, Tamil Nadu, India; 2 TVS-Sensing Solutions Pvt Ltd., Madurai 625122, Tamil Nadu, India; 3 Jothydev’s Diabetes Research Center, Trivandrum 695032, Kerala, India; 4 Madras Diabetes Research Foundation & Dr. Mohan’s Diabetes Specialities Centre, Chennai 600086, Tami Nadu, India; 5 SharkNinja, Needham, MA 02494, USA; 6 Departrment of Electrical, Computer and Biomedical Engineering, University of Pavia, 27100 Pavia, Italy

**Keywords:** Elderly type 2 diabetes (E-T2D), internet of medical things (IoMT), digital twin (DT), human digital twin (HDT), learning-based model predictive control (LB-MPC), personalization, precision medicine

## Abstract

Managing Elderly type 2 diabetes (E-T2D) is challenging due to geriatric conditions (e.g., co-morbidity, multiple drug intake, etc.), and personalization becomes paramount for precision medicine. This paper presents a human digital twin (HDT) framework to manage E-T2D that exploits various patient-specific data and builds a suite of models exploiting the data for prediction and management to personalize diabetes treatment in E-T2D patients. These models include mathematical and deep-learning ones that capture different patient aspects. Consequently, the HDT virtualizes the patient from different viewpoints using an HDT that mimics the patient and has interfaces to update the virtual models simultaneously from measurements. Using these models the HDT obtains deeper insights about the patient. Further, an adaptive patient model fusing this information and a learning-based model predictive control (LB-MPC) algorithm are proposed. The geriatric conditions are captured as model parameters and constraints while solving the LB-MPC to personalize the insulin infusion for E-T2D management. The HDT is deployed on and illustrated with 15 patients using clinical trials and simulations. Our results show that HDT helps improve the time-in-range from 3–75% to 86–97% and reduces insulin infusion by 14–29%.

## 1. Introduction

Type 2 diabetes (T2D) is characterized by reduced insulin secretion from the pancreas and is widely prevalent in low-and-middle-income countries, and also industrialized countries like the US [1]. Unlike type 1 diabetes (T1D), where patients are characterized by a complete lack of insulin secretion, in T2D relative insufficiency and/or inefficiency of insulin is common. Therefore, T2D requires continuous monitoring due to intermittent behavior and influences of certain factors: food, activity, etc., leading to frequent hyper and hypo events. This leads to many challenges in understanding and managing T2D (T2D). The problem becomes even more challenging in elderly people due to the changes observed with the aging, a.k.a geriatric conditions [2,3]. These geriatric-specific conditions are co-morbidity, multiple drug intake, reduced activity levels, pronounced diet influences, variability in nutrient absorption with age, hormonal changes, and other aspects. These factors evolve with age and progress as well, thereby making the blood glucose levels (BGL) highly unpredictable and intermittent. Similarly, insulin efficiency could also vary within a patient or among elderly patients with similar conditions. Therefore, personalization becomes the cornerstone for managing elderly T2D (E-T2D) effectively. In particular, precise insulin infusion considering the underlying geriatric conditions for managing E-T2D becomes important. However, this is cumbersome, as capturing certain geriatric factors to develop insights by itself is insufficient, and existing studies have not completely explored these specific aspects. This points out to an urgent need to propose a holistic approach for personalization that provides deep insights about various aspects and fuses them to decide the insulin dosing for BGL management.

### 1.1. Existing Methods

Traditionally, oral hypoglycemic agents (OHAs) and insulin infusion are two widely used approaches in managing T2D. The OHA in the form of pills is recommended for patients who are newly diagnosed or not insulin dependent. Nevertheless, over long time periods, these patients become insulin dependent as well with age or other factors [4,5]. Therefore, eventually, insulin administration becomes the treatment option for most patients. In such cases, insulin infusion could be either manual or automatic. In manual infusion, the patient or caregiver infuses the insulin based on the diabetologist’s recommendation. The interventions are intermittent and open-loop in the sense that the BGL is not continuously monitored. Therefore, conventional insulin therapy will have multiple challenges in those with advanced T2D.

Automated insulin delivery, also known as an artificial pancreas (AP), is touted as a solution for managing T2D [6]. The AP continuously monitors interstitial glucose level (IGL) by using a continuous glucose monitoring (CGM) sensor and infuses insulin to manage diabetes to be within 70–180 mg/dL (called the euglycemic range), using a control algorithm. Control algorithms for computing insulin dosing are the heart of AP systems. Different control algorithms for infusing insulin have been proposed both in practice and the literature. Existing control algorithms can be broadly discerned to be reactive and predictive. Reactive control action includes control algorithms such as proportional-integral and derivative (PID), fuzzy, and other approaches that compute insulin based on current measurements and lack predictive capabilities [7,8,9]. Some additional capabilities are available in the reactive controllers as well. For example, the Medtronic 722 has a PID controller with features such as automatic insulin shut-off when the BGL reaches closer to the lower target value and could include meal announcements in the insulin infusion plans. Still, these actions are based on measurements and not on patient-specific data, which is becoming a shortcoming in elderly patients. Predictive controllers, on the other hand, use patient models to study future behaviors and compute insulin infusion to manage T2D. The model predictive controller (MPC), a sophisticated control algorithm based on optimization routine and patient model, is revealed to be a promising solution [10,11]. This is mainly due to the fact that the MPC uses an approximate patient model to compute the insulin infusion and could embed real-time measurements in such computations. The role of an MPC to include aspects such as food influences and physical activity has been proposed in [12,13,14].

Though an MPC provides significant benefits and is suited for personalization, the need for patient models makes its design overwhelming. This is mainly due to model parameters whose computations require simulations or measurements (e.g., plasma insulin) that are difficult to obtain. Despite these difficulties, Omnipod 5 and Tandem t:slim X2 Control IQ are two successful MPC implementations available as commercial solutions [15]. Studies reporting on their safety and efficacy have been proposed in [16]. In spite of this success, patient-specific models and embedding aspects such as comorbidity still remain a challenge. Consequently, MPC implementation is relatively quite cumbersome to personalize for particular patients due to the need for computing model parameters. In practice, obtaining a patient model is difficult, and existing models rely on data such as plasma insulin that could be obtained from laboratory studies only. For this reason, simulators are being proposed and used for studying MPC efficacy. However, designing an MPC is limited due to the need for complex patient models (e.g., nonlinear), model parameters, optimization routines (nonlinear programming), and implementation in resource-constrained hardware (embedded processors with limited memory and processing power). This lends several challenges to personalization, and the focus is still open to obtaining models that are simple yet able to comprehensively capture BGL evolution, as this would also make optimization models simple and easier to be solved in embedded hardware.

Recent research is more focused on personalizing the AP [17]. To this extent, patient-specific models are proposed that capture certain intricate aspects (e.g., insulin–glucose interaction). A personalized AP for T1D with validation trials up to 6 months on individual patients has been reported in [18,19]. More recently, several long-period trials lasting months have been outlined in [20,21]. However, to our best knowledge, these personalization efforts are not oriented towards E-T2D management. This is mainly due to the fact that obtaining personalized patient models factoring geriatric conditions is rather cumbersome, and multiple models may be required that captures specific viewpoint and provide various facets to the patient model. Clearly, handling E-T2D requires novel approaches going beyond traditional ones currently used for managing T2D. Moreover, as various facets need to be considered continuously, a framework is necessitated in a complex model, and certain modules can be turned off as required—a general approach that is widely used. Further, such a framework requires capturing sensor and patient-specific data, knowledge creation from raw data, discerning patterns, understanding outcomes, and using it to manage as well as simultaneously personalize the E-T2D treatment. A framework is required for depicting the patient from a different viewpoint and also having capabilities to obtain the data required for orchestrating these different models. Notwithstanding this, to our best knowledge, a framework to personalize and manage E-T2D is completely lacking and has not been studied in the literature.

The digital twin (DT) is a framework that could fuse multiple models, data, and provide interfaces to virtualize physical entities with different viewpoints. In essence, the DT creates a digital replica of a physical object, process, or system orchestrated through a suite of models that exchange information with physical entities in real-time [22]. The DT was conceptualized around 2003 by Grieves for product life-cycle management in manufacturing, and it proposed a three-dimensional concept [23]. The concept was extended with five entities: physical object, digital representation, interconnections, data, and services [24]. The importance of the DT could be underlined by the fact that it is touted as one of the three emerging technologies in 2020 as per the IEEE Computer Society https://www.hpcwire.com/off-the-wire/ieee-computer-society-unveils-its-2022-technology-predictions/ (accessed on 2 December 2022). Major drivers for their proliferation are the Internet of Things (IoT), artificial intelligence (AI), machine learning, and big-data paired with digital and real-objects. The DT integrates these technologies appropriately for real-time data aggregation, system analysis, status monitoring, knowledge creation, providing deeper insights, risk management, bespoke displays, informed actions, agile situation management strategies, prediction, and others ([25,26,27] and references therein). Consequently, a generalized concept and notion for DT are still evolving. Notwithstanding this, the DT is proliferating other domains such as space cooling/heating [28,29], wind turbine fault diagnosis [30,31,32], bio-processes [33,34], energy [35], oil and gas [36], automotive [37,38,39], infrastructure [40,41], and defense [42] to name a few. More recently, DT concepts are being proposed in healthcare and medicine and are expected to provide significant advantages [43,44]. Mainly the DT is expected to personalize treatment pathways.

#### Digital Twin in Healthcare and Medicine

Human digital twins (HDTs) for healthcare are emerging as a cornerstone for personalizing treatments [45,46]. The prevalence of the IoT devices that can connect to user devices to collect, store, process, and notify in real-time through even mobile applications or embedded sensors is becoming a norm. By combining the patient replica with real-time data, it is possible to get deep insights into the patients’ conditions, manage conditions, predict future occurrences, monitor patients’ current conditions, and make informative decisions. Furthermore, simulations, dynamic models, and domain informed data-based models could help increase intelligence to manage, and alert users.

Amongst the relevant research in this domain, a reference model for DT healthcare was proposed in [44]. The framework enabled self-adaptation and autonomic computing for continuous monitoring and forecasting of chronic disease diabetes. However, process implementation was lacking. A cloud-based DT for elderly healthcare was proposed in [47]. Cloud-DTH was a reference framework that combined the cloud model with the DT model. This enabled individualized healthcare. However, the case studies presented lacked a performance evaluation and results illustrating this aspect. Karkara et al. [48] proposed DT for hospital systems using discrete-event simulation and IoT devices. Flexsim HC software was used to test the feasibility of the proposed methodology with different scenarios. However, the DT has not been explicitly brought out in the investigation.

Detecting seizures before they surface using machine learning models has been presented in [49] through data collected from drug-resistant epilepsy patients. The authors proposed a deep-epileptic seizure detection using a surrogate gradient descent-based approach. However, complexity and implementation aspects have not been studied. Personalized healthcare using an HDT for personalized healthcare has been reviewed in [50]. The HDT is a new direction in research that provides tools to provide proactive, prescriptive, accurate, and efficient personalized healthcare system (PHS) to patients using DT concepts.

The HDT has three parts: patient (PT), virtual digital twin (VT), and PT–VT interactions. However, orchestrating an HDT requires novel data sources, new models, and techniques to achieve personalized management. The main advantage of an HDT is its ability to handle both historical and real-time data that is missing in current approaches. With an HDT, management could be tailored to focus on a particular individual’s contextual data. The role of digital twin technology for Healthcare 4.0 was studied in [43].

Existing works clearly point out an HDT’s ability to personalize treatment by combining various data sources, models, and techniques. An HDT could leverage data to optimize patient-specific treatment plans, recommend life-style modifications, improve management, and reduce overall costs incurred by E-T2D patients. Furthermore, an HDT provides prescriptions by predicting future patient states and preventing hyper and hypoglycemic events. However, there exist some pertinent questions to be answered for successful HDT-based personalized E-T2D management. These questions include whether underlying influences on E-T2D, such as comorbidity, multiple drug intake, diet influences, activity levels, and other patient-specific aspects, could be used for E-T2D management and whether inter- and intra-patient variations could be detected using temporal data patterns to provide deeper insights. In addition, extracting precise medical data and techniques to obtain models that could capture aspects that are contextual to a particular patient needs to be evolved.

Our objective is to illustrate the role of an HDT in managing E-T2D through patient virtualization from different perspectives for delivering precision insulin. However, this requires personalization and modeling different aspects and fusing various patient-relevant data. To this extent, the HDT builds a suite of models to gain deeper insights on the patient. These models exploit the raw data and convert them to knowledge by mining them. The HDT’s ability to exploit historical, contextual, image, and other forms of data to create deeper insights about the patient becomes a major enabler for precision insulin. Finally, to prove an HDT’s ability to manage E-T2D, clinical trials to generate patient models and simulation studies to show that precision insulin delivery are used as illustrations. Our main contributions are:

(i) A new HDT framework and architecture towards personalizing E-T2D management with capabilities to aggregate data, a suite of models that build intelligence on the data, and an interface between the VT and PT.
(ii) An IoMT architecture to aggregate data vis-á-vis HDT for E-T2D management.
(iii) Modules for forecasting, food nutrient predictions, time-series trending, and other intelligence required for managing E-T2D.
(iv) An adaptive patient model that personalizes insulin infusion based on geriatric factors and learning-based MPC (LB-MPC) that could embed the deep-learning models to compute precise insulin infusion.
(v) Illustrate the HDT’s capability to manage E-T2D by modeling a personalized patient model and embedding other aspects. To this extent, clinical data from patients are collected for 14 days to obtain patient model and patient-specific contextual data from 15 elderly patients. Using these models and data, simulations are performed to illustrate the HDT’s ability to deliver precision insulin considering various aspects.

The paper is organized as follows: Section 1 introduces the concepts. Section 2 presents a detailed description of the HDT framework architecture, and its components. Section 3 presents the HDT implementation aspects for the various modules. Section 4 presents the clinical trial and simulation results. Section 5 summarizes the conclusion and future course of the investigation.

## 2. HDT Framework Architecture

The paper proposes and implements an HDT framework for context-aware E-T2D to enhance management and achieve deeper insights. The proposed HDT framework uses IoT devices and sensors to aggregate data from elderly patients using an IoMT architecture. Similarly, it builds a suite of models to capture different aspects of the E-T2D patient. The models can be domain informed data-based models (e.g., machine learning), dynamical models based on differential equations, or hybrid models that can represent a complex nonlinear dynamical model of the elderly patient. Such models can capture specific aspects of the patient. The tools along with the IoMT architecture, create a virtual replica of the E-T2D patients and help to manage BGL within safe bounds. Furthermore, it enables learning inter- and intra-personal variations by cooperating to learn data from similar patients and within the same patient. It is to be noted here that such data is more frequent and has higher data rates than typical clinical measures. This helps healthcare professionals, patients, and caregivers to gain more insights to manage E-T2D.

Three basic entities of the HDT framework are Patient (PT), Virtual Twin (VT), and interfaces. The PT is the patient who needs to be virtualized by using historical data, sensor measurements, contextual data, images, and other data sources. The VT is the digital replica of the PT that could be used for managing E-T2D. Interfaces map PT to VT and transfer data (e.g., sensor measurements) or inferences (e.g., food influences on T2D). The PT and VT need a reliable interface that enables the co-evolution of both the PT and VT. Together PT, VT, and interfaces offer personalized healthcare services for E-T2D.

### 2.1. HDT Framework Components

**Figure 1:**
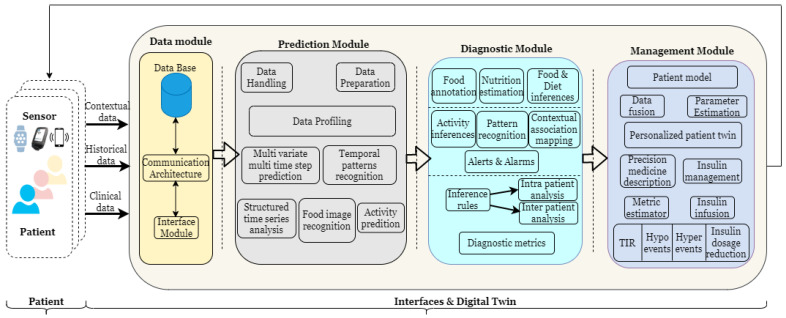
HDT architecture.

The HDT framework shown in Figure 1 has four functional modules: (i) a data module that is responsible for aggregating the current data, (ii) a prediction module that provides future values based on historical and current data, (iii) a diagnostic module that uses current measurements and predictions to discern actions to predict inter and intra-patient variations and provides explanations for certain decisions and conditions, and (iv) a management module that personalizes insulin infusion and maintains BGL within bounds, whereas a module is a sub-block within the entity performing a particular task or providing a service.

### 2.2. Data Module

The data module has devices to communicate, receive, store, and retrieve sensed data. To aggregate sensor data, an IoMT architecture is used to fuse IoT data with healthcare. Using IoMT architecture, vital signs, blood glucose levels, annotated food images, activity, and other sensed information are obtained. In addition, patient-centric contextual data such as diabetes history, family history, co-morbidity, drugs or multiple drug consumption, average calorie intake per day, and other data are collected. These data are aggregated and stored in the database in the data layer. The sensors are connected to the edge node and interfaced with the cloud. The database (DB) that contains all the patients’ data can be internal or external. Data can be stored, retrieved, and used from the DB. The details of the IoMT architecture, communication protocols, and communication services are discussed in the next section.

### 2.3. Prediction Module

The prediction module uses the sensed information and historical data as inputs and provides future predictions of various aspects. Critically, providing predictions requires accurate data. However, aggregating accurate data with IoMT architecture and devices is challenging. This is mainly due to the fact that IoMT uses compact sensors and modules that have limited computing and memory power. Therefore, methods for data handling and preparing the data for extracting additional features to be used for predictions are required. The predictive module components—data handling and data preparation—are used to this extent.

The data-handling component uses data imputation methods such as filling average values, past samples, or future samples. To this extent, it uses mathematical tools such as auto-correlation, partial auto-correlation, variance, and standard deviation to test the accuracy of the data imputation method. Second, the data preparation components provide scaling and feature extraction and study statistical aspects, such as average, minimum value, maximum value, variations from maximum to minimum, and others. These statistical measures provide valuable insights into individual patients and are important for E-T2D management and the detection of anomalies.

The data profiling component uses a set of rules to provide the data required for prediction algorithms. The rules are implemented in the framework depending on the data to be used as input to a particular prediction algorithm. Temporal data predictions and pattern recognition from input data are required for understanding geriatric conditions’ effect on the BGL. In addition, the structural time-series component is a prediction method used to understand periodicity, cycles, average value, trend, and other information that could be obtained, which is critical for making predictions of the future states of the patient. Food image recognition obtains food images and recipe data to understand the nutrient intake of a particular patient. This information is used to understand the food influences on the BGL, and it estimates the carbohydrate (CHO) intake from the data. The level and type of physical activity (e.g., standing, sitting, and walking) detection are done by using the sensor measurements. The sensor tag is used to this extent. A detailed explanation of the activity detection is avoided here, considering the focus of the paper, and readers are referred to [12] for further data.

### 2.4. Diagnostic Module

The diagnostic module provides valuable insights from the data module and prediction module. It has components that crunch predictions and measurement data to provide valuable insights. The diagnostic tool can provide insights into time-series patterns based on influencing factors or could even explain individual samples and their outcomes. To diagnose patterns, a matrix profile-based analysis is used, and to detect outcomes from individual samples, explainable artificial intelligence techniques are employed. These tools provide capabilities such as motif discovery, explanations of hyper and hypo conditions, and others. Similarly, the diagnostic tools have inference rules that help detect time-series patterns of different events.

Similarly, to detect CHO and food nutrient-related aspects, queries are added to food images, and annotations are created for the images based on user feedback. This helps overarching current food image processing tools with feedback from the user. Consequently, nutrient predictions and their accuracy could be improved. The diagnostic module performs this action. Considering the brevity of the paper, discussions on food image processing are avoided here. The output of the diagnostic tool is the input to the management module.

### 2.5. Management Module

The management module aids precision medicine by making suitable recommendations to physicians and caregivers. This way, the module helps personalize the insulin infusion, food recommendations, activity levels, and other aspects. Data fusion algorithms combine inferences from the personalization component with a personalized patient model to compute model parameters. However, these parameters capture the influences of the patient-specific aspect that could change over time. An adaptive patient model captures the dynamic influences of geriatric factors on patients. The personalized patient twin is obtained from the parameter estimation component. Here the contextual data is also embedded in the computation. Using the personalized E-T2D management twin, personalized insulin infusion is computed using the management component to maintain BGL within specified bounds. The metric estimator is used to compute time-in-range, hyper and hypo events, model errors, or other such factors. Insulin dosage reduction and other metrics can be evaluated using the time spent in hypo, hyper, and Time in Range (TIR) conditions

**Figure 2:**
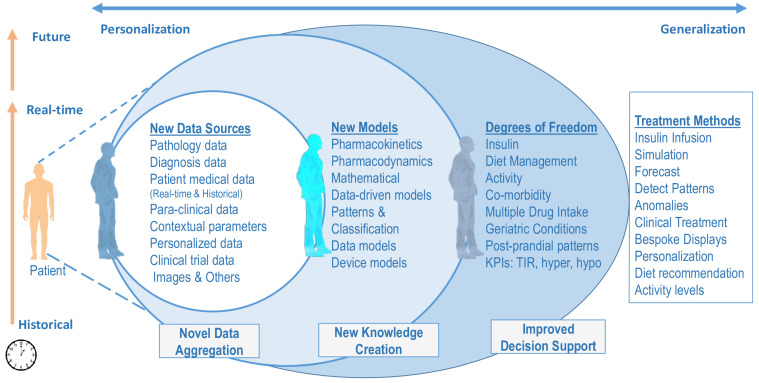
Precision Treatment Methods Through HDT—The HDT framework leads to new data, novel models that create additional knowledge, and improved decision support through new degrees of freedom.

The HDT framework presented above leads to new data, novel models that create additional knowledge, and improved decision support through new degrees of freedom. The three aspects are illustrated in Figure 2. Different components of HDT enable new data streams, novel models that use these data, and new variables that could be used for managing E-T2D. However, for the rest of our analysis, the treatment method is based on precision insulin infusion alone. Other aspects, such as behavioral interventions, are not considered in our analysis.

## 3. HDT Implementation

This section describes the HDT implementation aspects. Detailed discussions on the components and their realization are provided in this section.

### 3.1. IoMT for Data Module

**Figure 3:**
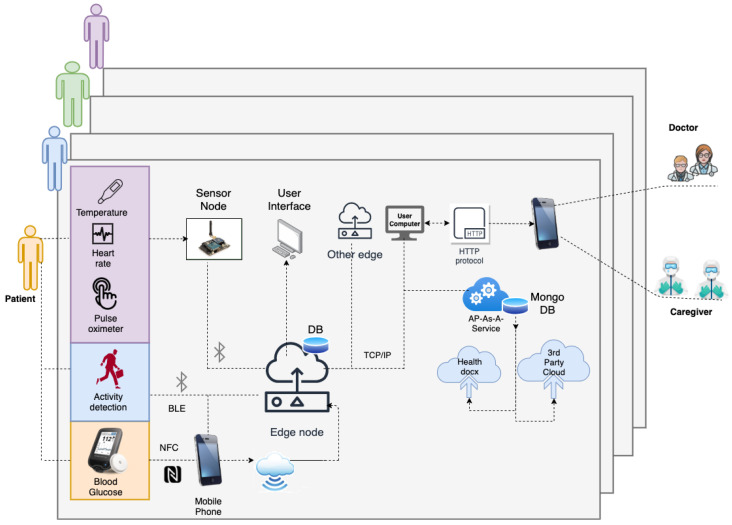
IoMT Architecture—Data transfer using IoT sensor devices from patients’ (left), mounted devices to edge nodes (center) to the HDT architecture and finally to medical personnel and caretakers using a mobile app.

The IoMT blends medical devices with the IoT as depicted in Figure 3. Medical devices have different communication requirements, such as personal area network (PAN)-based communication for transmitting patient data. Protocols such as Bluetooth low energy (BLE), near field communication (NFC), or bluetooth are examples of PAN. These devices generally transmit information reliably only over small distances. However, this information needs to be transmitted to longer distances for processing. Therefore, edge nodes are required to connect to PAN and be able to transmit the data to the cloud or other platforms. The edge nodes connect with sensors on one hand and have WiFi connections to connect with cloud platforms on the other. Further, as wearable sensors are commonplace today, the IoMT architecture should be flexible enough to integrate them on-the-fly. Measurements collected from IoMT architecture include vital signs, blood glucose levels, or other physical data about the patient. Moreover, HDTs require cloud services for data collection, and the IoMT architecture requires these capabilities. In what follows, the IoMT architecture and its components are discussed.

The IoMT architecture for HDT has three layers: the perception, transfer, and application layers. The perception layer has compact sensors to measure BGL using continuous glucose sensors that use NFC and transmit the data to a mobile app. In addition, physical activity is detected through an accelerometer, gyros, and wifi probes, along with vital signs: (heart rate and blood oxygen saturation level) using MAX30100 based monitor, temperature (DS18B20). For detecting activity, Texas Instruments’ sensor tag CCS2210 is used. It is placed on the patient’s hip through suitable arrangements to prevent vibrations that cause noisy measurements. The sensed information is collected by the sensor node using RF and/or WiFi communication. The NRF24L01 and RFM69HCW modules are used for RF communication. The BGL is monitored using the FreeStyle Libre Pro sensor, which sends the IGL readings to a mobile app using the NFC protocol. The sensing layer is limited in communication range, and the sensor data needs to be communicated to the cloud to off-load HDT computations.

The transfer layer is used for off-loading. The transfer layer has the edge node; a Raspberry Pi 3 is used in our case. It communicates with the mobile app through WiFi to collect data. The mobile app collects the data using NFC and BLE interfaces. Vital signs, BGL, activity levels, and other information are transmitted to the mobile app using the BLE. The mobile app uses WiFi connection to transmit the data to the edge node. Inside the edge node, a small database is created using Python scripts to read, store, and retrieve data every 15 min. In addition, the edge node can communicate with other edge devices and the cloud via WiFi interfaces. The patient, doctors, caregivers, and other stakeholders can connect to web interfaces to view their data in real-time or historical patterns. The IoMT architecture proposed in this work is shown in Figure 3.

**Figure 4:**
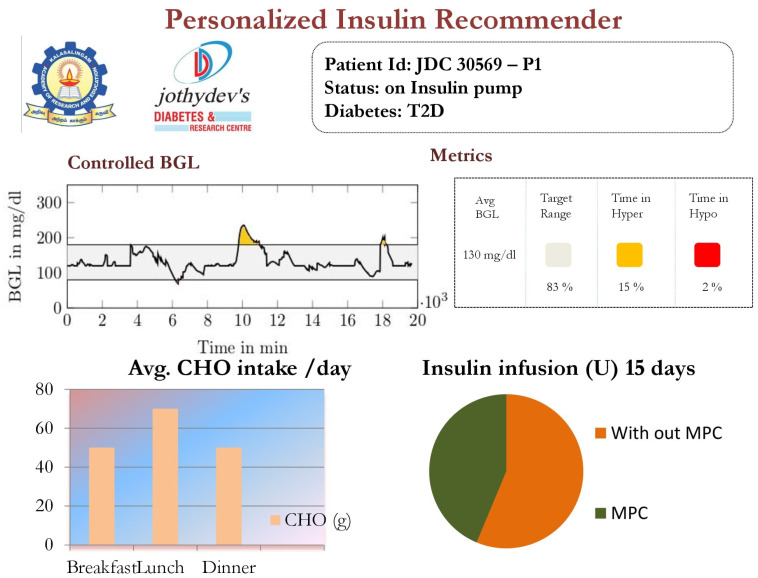
Simulated web page view of the patient’s data.

The edge node also provides various services. These include hardware interface services, monitoring services, hosting software (back-end and front-end), HDT-related services, and cloud-based services. These services are orchestrated through a back-end realized using Python scripts. In our implementation, our UI is created with Flask, a Python based front end development tool that updates hypertext modeling language (HTML) files. It uses the Jinja2 notation for coding the HTML files. The Flask application is used to connect the front end with the back end that implements the services required for managing the E-T2D interfacing with the cloud. A typical UI with data visualization is shown in Figure 4.

**Figure 5:**
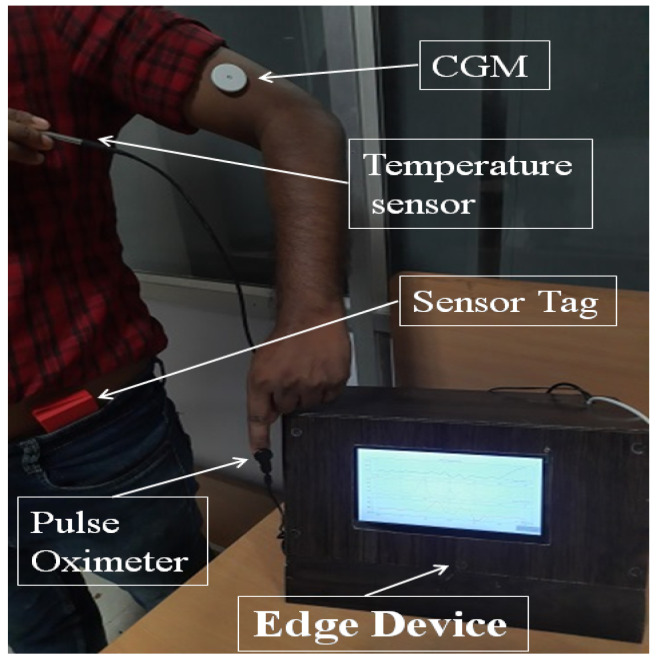
Real-time patient data collection system using temperature sensor, pulse oximeter, CGM, sensor tag, and Edge device to display the data.

Similarly, the edge-node has an auto-device discovery system through which sensors are connected to the edge devices. Even wearable sensors could communicate to the edge node through BLE connections. Device discovery is part of the monitoring services. As each sensor has a specific UUID for configuring and connecting to specific services in the IoMT, not only device discovery but mapping to services could also be achieved. The edge node with sensor and communication interfaces are shown in Figure 5. Each such edge unit is deployed for the patients. The IoMT layer provides the interface to update the VT model for the HDT. In addition, calibration, and device configuration are additional services provided by the monitoring services in the edge node. The storage extension services offered by the back-end manage the local MySQL database in the edge node and MongoDB database ported to the cloud using Healthdocx, a cloud platform.

The application layer implements the actual HDT in the edge node, and a virtual version is available on the cloud. The back end uses the raw sensor data and transmits it to the cloud to provide various insights and insulin recommendations to the patient factoring in various personal aspects (e.g., co-morbidity). The different modules performing computation and the application layer of the IoMT architecture are discussed in the next section.

### 3.2. Prediction Module

The prediction module has the following components (see Figure 1): (i) Time-series forecasting, (ii) Food image recognition algorithm, and (iii) Structured time-series analysis. These components use patient contextual data, clinical time-series data, and others to predict future blood glucose evolution.

#### 3.2.1. Multi-Time Step and Multi-Variate Time-Series Prediction

As stated earlier, blood glucose levels in elderly patients are influenced by various geriatric factors. Understanding future BGL variations requires predictions using these factors. Accurate prescriptions for personalized insulin to manage diabetes requires a BGL forecast. Moreover, a forecast is required for multiple time steps in the future. Consequently, multi-variate and multi-time step forecasting is required for predicting BGLs. However, time-series forecasting for multi-variate and multi-time steps is quite different from other regression models. First, the data sequencing and ordering are important. Second, multi-variate and multi-time step forecast with conventional mathematical methods is rather difficult. Third, patient-specific factors may introduce nonlinear and time-varying behavior.

Existing approaches for time-series forecasting: auto-regressive moving average, auto-regressive integral moving average, auto-regressive exogenous, etc., lack the capability to handle multivariate and multi-time step models. These models work when there is a strong correlation between the current value and past sequences. However, with sudden food intake or insulin dosing, the influences can be varied. Artificial neural network (ANN) models could model time-series data. The recurrent neural network (RNN) is a widely used method for time-series predictions. The RNNs predict the future output based on past measurements and past outputs. Therefore, there is inherently a feedback mechanism. Nevertheless, RNNs lack the capabilities to handle long-term dependencies, as only past input is used as feedback. Further, their short memory makes them unsuitable for time-series forecasting tasks. Besides, the RNNs lack control over past samples and their relevance. In other words, the RNNs lack the capability to forget past samples whose contribution towards current prediction is low. Other computational issues, such as exploding and vanishing gradients, make them unsuitable for multi-time step and multi-variate forecasting.

The Long–Short Term Memory (LSTM), a deep-learning model, is designed to overcome vanishing and exploding gradient issues with RNNs [51]. Further, LSTM can handle long delays, noise, and data loss, and has more degrees of freedom to tune the network. Yet, LSTM-based multi-time step and multi-variate forecasting are still not fully realized. This is primarily because current predictions have to be carried forward for computations and are feedback as well. Therefore, error propagation happens both in forward and feedback paths.

Our idea is to use LSTM for forecasting blood glucose levels for multi-time variate and multi-time-steps. The inputs to the LSTM model are insulin infusion rate, food intake as carbohydrate (CHO), and past time samples of blood glucose levels. The output is the future blood glucose levels for the next 1 h. The LSTM captures the long-term temporal correlations towards predicting the BGL variations for a pre-defined duration (1 h) and sampling rate (15 min).

In essence, an LSTM is a recurrent neural network capable of sequence learning, which combats the vanishing gradient problem that is characteristic of traditional recurrent neural networks. This is achieved through a set of gates including input gates, forget gates, and output gates. Thus, given a time series sequence $\left\{s_{1},\ldots ,s_{t},\ldots s_{T}\right\}$, the objective of LSTM is to learn the temporal dependencies in $\left\{s_{1},s_{2},\ldots ,s_{t-1}\right\}$ to predict $s_{t}$. The responses of the various gates are given by,

(1) $$\begin{array}{ccc} i_{t} & = & \sigma \left(w_{i}\left[h_{t-1},s_{t}\right]+b_{i}\right) \end{array}$$

(2) $$\begin{array}{ccc} f_{t} & = & \sigma \left(w_{f}\left[h_{t-1},s_{t}\right]+b_{f}\right) \end{array}$$

(3) $$\begin{array}{ccc} o_{t} & = & \sigma \left(w_{o}\left[h_{t-1},s_{t}\right]+b_{o}\right) \end{array}$$

The hidden layer response of the network for the sequence $s_{t}$ is then given by

(4) $$h_{t}=o_{t}*\tanh\left(c_{t}\right)$$

where $o_{t}$ is given by Equation (3), and $c_{t}$ is the memory in the network that is given by

(5) $$c_{t}=f_{t}*c_{t-1}+i_{t}*\left(\tanh\left(w_{c}\left[h_{t-1},s_{t}\right]+b_{c}\right)\right)$$

where $w_{c}$ and $b_{c}$ are the weights and biases for the memory gates, respectively. The memory in the network ($c_{t}$) helps in capturing the long-term dependencies to remember the past. Thus, time-series data could be represented by LSTM through multiple gates, and it holds the temporal dependencies in the patients’ time-series BGL data [52,53]. To handle data loss the estimate from LSTM for the current time-instant is used as inputs.

**Figure 6:**
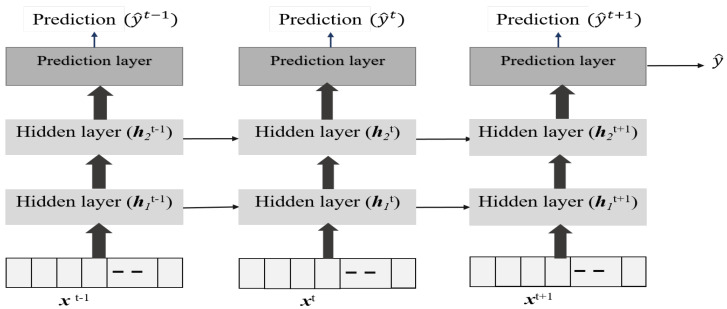
Structure of the LSTM Network for Blood Glucose Level Predictions.

**Figure 7:**
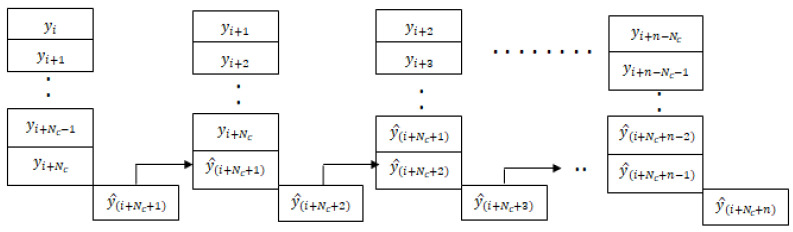
BGL prediction using time segmentation.

As shown in Figure 6, the LSTM model for the blood glucose estimator has two hidden layers, and it is unrolled in time to represent its sequence representation ability, as shown in Figure 6. One can observe that output at each time point could be derived in addition to the final time step. We use the past data over an observation window of 2 h to make a prediction after 15 min for 1 h (4 samples) or more time steps. Thus, the LSTM model represents the temporal correlations and helps us to predict BGLs after 1 h in advance so that this can be used to handle data loss and physiological delays with existing CGM and provide forecasting capability to the conventional BGL sensor. Figure 7, shows the flow of the LSTM prediction module, where y and $\hat{y}$ represent the BGL measurements and predicted value, respectively. Besides, $N_{C}$ denotes the prediction horizon. $y_{i},y_{i+1},y_{i+2},\ldots ,y_{i+N_{C}}$ samples are used to predict the BGL value ${\hat{y}}_{i+N_{C}+1}$. The predicted BGL value ${\hat{y}}_{i+N_{c}+1}$ is given as input to the next series to predict the BGL value. We denote such forecasting through predictions carried from the current step as time-series segmentation. Here, $N_{c}$ is used as the prediction horizon with abuse of notation to denote that this differs from the conventional prediction horizon of patient models.

The LSTM is trained on 70% data omitting 4200–6000 min. Then during the testing phase, the remaining 30% is used as with the usual approach used in the literature. Our LSTM model could forecast multiple time steps ahead based on multi-variate factors, and the forecasting step is repeated again during each horizon. This means that the forecast is corrected at each step. This is a multivariate and multi-time step LSTM with a moving horizon strategy. This means that in the current step, future time-step values are predicting incorporating recent measurements, and the procedure is repeated. The trained model was used to predict the final 30 % of the data. The performance metrics such as prediction error range, RMSE, and MSE for the blood glucose estimator, were computed using the test data, which is shown in Table 1, it shows that the LSTM can predict the BGL with an error percentage of 3.06–5.16% for the patients.

**Figure 8:**
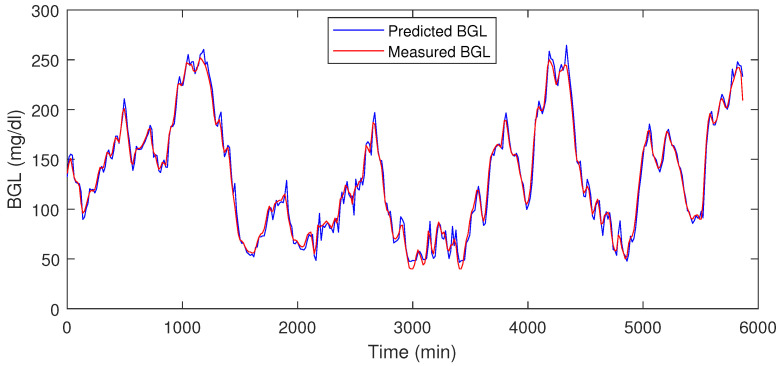
LSTM prediction result for P1.

**Figure 9:**
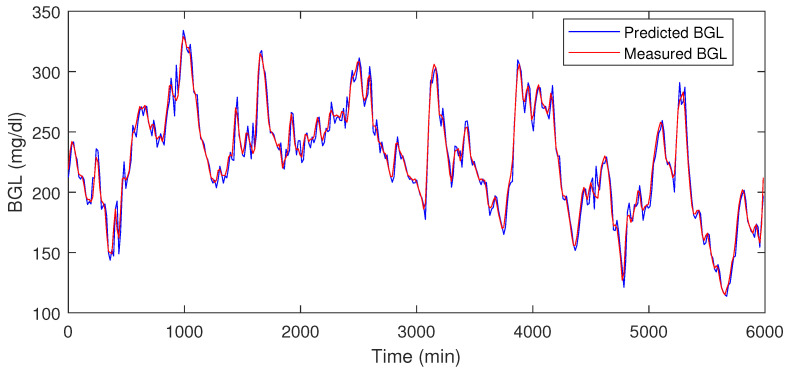
LSTM prediction result for P2.

Table 1 shows the prediction error % for the test data, and in Figure 8 and Figure 9 the red traces show actual measured values, blue traces show predicted value in a receding horizon manner as illustrated earlier. This means that the one-ahead sample that was estimated is plotted, and the rest are discarded. The procedure is repeated during each time-instant.

The LSTM-based BGL forecasting tool needs to be deployed in the HDT framework. This means the model needs to be stored and provide an output when one sample instance is given as input, and output is obtained for the next few hours. In our framework, the model was stored as JSON files automated using existing packages, and then when instantiated, the  forecast was provided from the model. The model is stored in JSON files as weights and through model callback functions, the forecasts are provided as output.

**Note 1:** As with BGL forecasting, our HDT model also uses food nutrient estimation using AI tools. The idea is to use food image recognition and using annotations as a feedback mechanism to improve the food nutrient estimation accuracy. However, the complete module description is beyond the scope of this paper, and our assumption is that food nutrients are available either through a food atlas or with a nutritionist’s recommendation. However, in the implementation, a food nutrient estimator could be implemented and has not been presented here, considering the focus of the paper.

#### 3.2.2. Structured Time-Series Analysis

**Figure 10:**
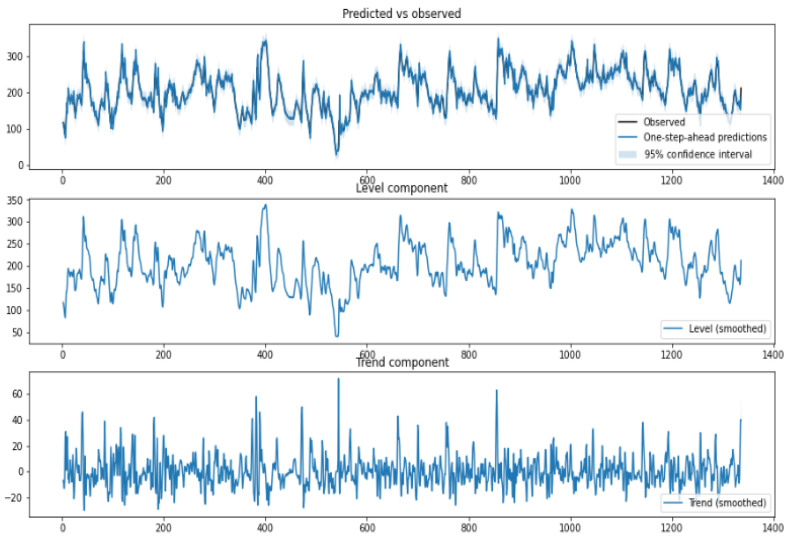
Structured Time-Series Analysis—The y-axis in the upper panel is BGL (mg/dL), and the time interval is 15 min.

The structured-time series analysis is based on an unobserved components model (UCM). The idea is to decompose the time-series data into trend, seasonal, cycle, and regression components. Leading to a linear decomposition to understand the time-series data. The structured time series provides a way to analyze time-series data for trends that are useful for capturing aspects such as hyper and hypo conditions. In our HDT, the structured time-series analyzer and miner module use the time-series data on BGL, insulin, and food to inform trends, cyclic factors, seasonal term (daily/weekly) variations, and errors. In addition, it also provides variance in these parameters. The generalized UCM is given by,

(6) $$y_{t}=\underset{\mathrm{trend}}{\underset{︸}{\mu_{t}}}+\underset{\mathrm{Seasonal}}{\underset{︸}{\gamma_{t}}}+\underset{\mathrm{cycle}}{\underset{︸}{c_{t}}}+\underset{\mathrm{explanatory}}{\underset{︸}{\sum_{j=1}^{k}\beta_{j}x_{jt}}}+\underset{irregular}{\underset{︸}{\varepsilon_{t}}},$$

where $y_{t}$ (BGL versus Time) is the outcome at time instant *t*, $x_{t}$ is the input vector, $\beta_{j}$ is the explanatory variables as in a linear regression, and $\varepsilon_{t}$ is the irregular component. Typically the trend component follows a normal distribution $\mu_{t}∼N\left(0,\sigma^{2}\right)$ wherein $\sigma^{2}$ models the variance. The trend in our analysis captures both (level and trend). Trends model the slope of the series in the absence of any other influencing variable. The UCM in our analysis is locally linear and has a slope term (positive or negative). The seasonal component in our analysis models the daily variations, and as the data samples are collected once in 15 min, the seasonal component is modeled for 96 samples. This means a daily seasonal component is considered in our analysis. The cycle component captures the cyclical aspects at time frames much longer than the seasonal component. The time-series components and their variance provide useful insights into inter and intra-patient variability. A snapshot of the structural time-series model is shown in Figure 10.

The prediction tools presented in this section provide valuable forecasts and estimations on certain food and time-series evolution. To interpret patterns and their outcomes, diagnostic tools are required. The next section presents the diagnostic module used in our HDT.

### 3.3. Diagnostic Module

The diagnostic module uses multiple tools to draw inferences based on data and prediction modules. Here, we illustrate the inferences from the time series that could be drawn from such analysis. We use motif discovery and anomaly detection as examples.

Motif Discovery

Time-series motifs are sub-sequences of longer time series that have similar patterns. Motifs indicate patterns that get repeated or the presence of similar patterns between two time series. Using motifs, dictionaries of recurring series could be created that would provide more insights into inter-patient and intra-patient variations. The motif discovery methods in the literature are based on the sub-sequencing technique wherein certain time-series samples are grouped and compared with similar sub-sequences. A quadratic metric, usually their Euclidean distance, is measured to see whether the sub-sequences are similar. A matrix constructed with the euclidean distances between sub-sequence is called the distance matrix, and a matrix profile (MP) is a vector that stores Euclidean distance between any sub-sequence within a time series and its neighbor [54]. The MP can be used to study patterns (motif), detect anomalies, and discover shapelets, time-series chains, and other structures that discover patterns in the time series. Further, MPs are used to detect anomalies that could alert to possible wrong sensor readings or abnormal conditions, an important aspect for building robustness in the system.

**Figure 11:**
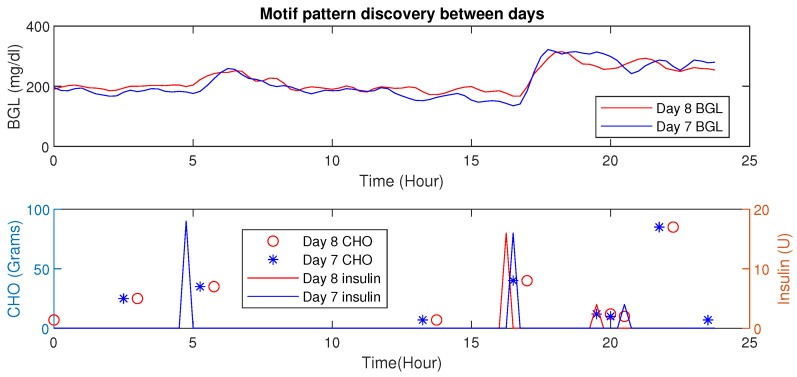
Time-Series Analysis: Motif Discovery between days of a P1. (Top) Dependent variable (BGL) versus time whereas the bottom show independent (controllable) behaviors (insulin) and CHO in food.

Using MP, similar patterns or periods having similar conditions (e.g., post-prandial periods with similar CHO intake) could be detected. Further, changes in Euclidean distances also reveal valuable information about the impact of influencing factors (e.g., activity signature on the BGL evolution). The inter-patient motif discovery in patient $P_{1}$ is shown in Figure 11. It is evident that for similar CHO and insulin infusion profiles, the BGL patterns are similar. The Euclidean distance computed in this case is 2.01 (mg/dL in minutes), indicating similar patterns.

**Figure 12:**
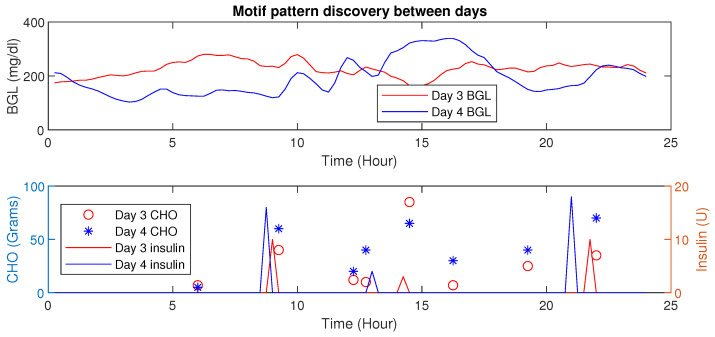
Time-Series Analysis: Motif Discovery between days of P1—The upper panel shows a dependent variable (BGL) versus time whereas the lower panel shows independent (controllable) behaviors (insulin) and the dependent variable CHO.

An increase in Euclidean distance is observed for dissimilar patterns as shown in Figure 12. Such analysis provides the ability to discern different conditions.

More detailed results with diagnostic tools based on time-series are presented in the results section. By identifying similar patterns, the patient can understand influences such as activities on BGL evolution. Still, patients require certain influencing factors posterior to their actual outcomes. The explainability of the hyper and hypo conditions is quite important in diagnosis and is the focus of the next section.

Explainable Diagnostics for Events

Another aspect of the diagnostic tool is the explanations provided for hyper and hypo events through explainable artificial intelligence (XAI). While a matrix profile-based analysis provides ways to explain patterns, individual samples causing a particular outcome are explained using XAI.

The XAI is a technique to explain decisions to stakeholders. The Local Interpretable Model-Agnostic Explanations (LIME) [55] is a tool that explains single data instances being model agnostic. Explanations generated by LIME for data instance *x* are defined as:

(7) $$\mathrm{explanation}\left(x\right)=\arg\min_{g\in G}L\left(f,g,\pi_{x}\right)+\Omega \left(g\right)$$

where f() is the model, g() is the local explanation for instance *x*, and *L* is the loss function that measures the fidelity between f() and g() while keeping the model complexity denoted by ω(g) low. The LIME uses a neighborhood $\pi_{x_{*}}$ of the argument ${\underline{x}}_{*}$ in which approximation is sought. In general, g() denotes a class of interpretable models, G; such models could be decision trees or other simple linear models.

Our explainable diagnostic tool has two components: the base-learner model and eXplainable Artificial Intelligence (XAI). The eXtreme Gradient Boost (XGBoost) classifier is used as the base learner, and the locally interpretable model agnostic explanations (LIME) tool is used to explain hyper/hypo events that are acquired using the data-instance profiler module. The XAI tool explains the factors that lead to hyper and hypo events in a patient, thereby helping personalization and improving patient behavior by highlighting behaviors that lead to hyper- and hypo-events. The results section shows explanations provided on single data points using XAI.

### 3.4. Personalization and Management Module

This section describes the details of the E-T2D management module.

#### 3.4.1. Adaptive Personalized Patient Model

The personalization module is responsible for precise insulin infusion, maintaining BGL accurately within bounds, and handling patient-specific aspects. To handle BGL variations due to geriatric factors, a semi-parametric regression model is used. The basic idea here is to have an adaptive parameter estimation algorithm where the patient-specific parameters are identified using current measurements and predictions. The geriatric factors are embedded using parameters that denote these aspects, as explained in this section.

We present the personalized patient model and personalized insulin computation algorithm based on the model predictive control technique. Our model is an adaptive model that is learned from data samples, and the personalization algorithm infuses insulin based on individual conditions. However, time-varying and nonlinear behaviors would require an adaptable model whose parameters change with time. Such changes also explain the influences of factors such as diet on the patient. Therefore, a patient model that is simple and adaptable needs to be proposed. This section presents an adaptable patient model that helps manage E-T2D toward building a personalized HDT [50]. The personal glucose dynamics is described as

(8) $$G\left(k+1\right)=\zeta_{1}G\left(k\right)+\zeta_{2}I\left(k\right)+\zeta_{3}CHO\left(k\right)\;$$

The model in (8) captures the patient’s complex dynamics by considering the influences of food and insulin.

(9) G(k+1)=H(k)w(k)+ε

H(k)=[G(k)I(k)CHO(k)]

$$w\left(k\right)={\left[\zeta_{1}\;\zeta_{2}\;\zeta_{3}\right]}^{T}$$

where ϵ is assumed to be normally distributed bounded additive noise with $∥\left(\varepsilon +\varepsilon_{e}\right)\left(k\right){∥}_{2}\leq \alpha$ with $\alpha \in R^{+}$.

(10) $$\begin{array}{cc} \underset{w(k)}{min}\sum_{k=0}^{N_{P}}\lambda^{N_{P}-k} & {[\left(G\left(k+1\right)-H\left(k\right)w\left(k\right)\right)}^{T}\Pi \left(k\right)(G\left(k+1\right)\\ \; & -H(k)w(k)]\\ \; & s.t.\;\;\;\;\;\;\Pi \left(k\right)\in R^{+} \end{array}$$

In the recursive model parameter estimation, at each time instant *k* (*k* is the time step for 15 mins), the optimization problem in (10) is solved to identify the parameter with forgetting factor λ. Note that the forgetting factor might be adjusted for each patient. The model adaptation steps are shown in Algorithm 1. The initial input to the algorithm is inverse correlation matrix *P* and the forgetting factor λ. Once the new measurements from the patient are obtained, the model will predict the BGL, and the algorithm checks the difference between estimated and current BGL measurements, if there is an error, then the parameters will be updated; otherwise, it keeps as same as the previous. The algorithm uses the new measurements to adjust the model parameters w(k).
**Algorithm 1:** Dynamic Patient Model Parameter Estimation for BGL Prediction.
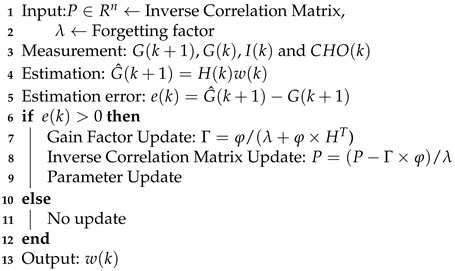


**Figure 13:**
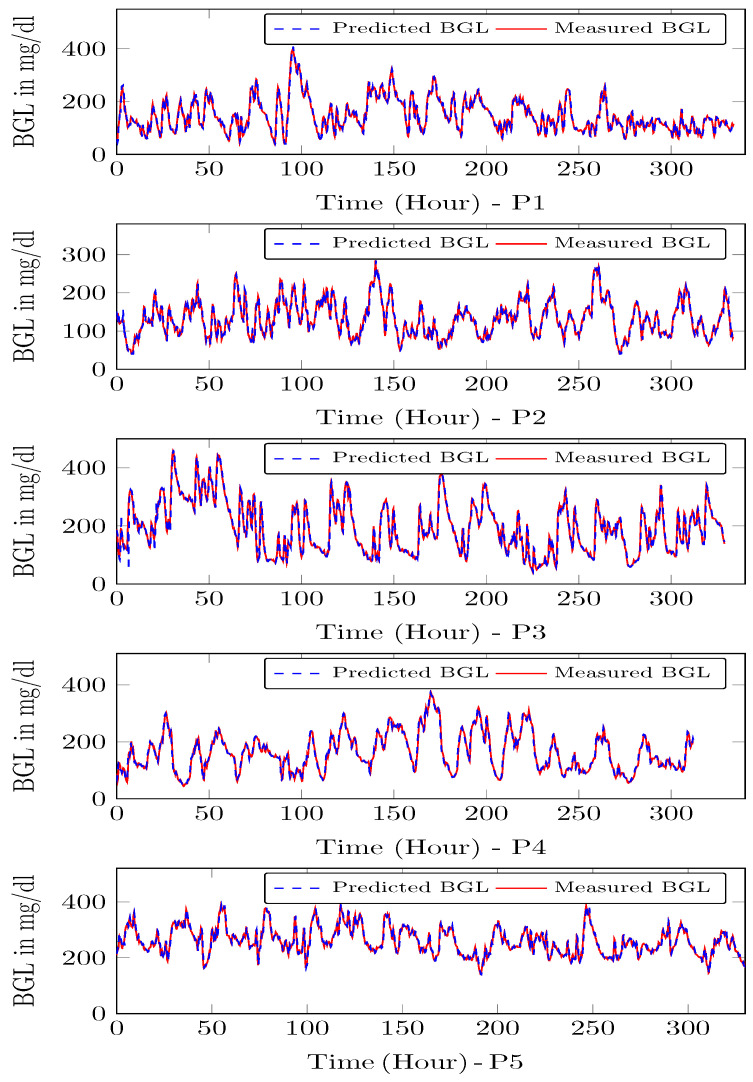
Model predictions for P1–P5—The time step is 15 min, and the BGL is predicted for one step ahead.

The adaptive model updates the parameters every computation time, i.e., 15 min. So first, the model is updated and used to compute the insulin infusion for the next hour, and the process repeats. During each time-instant, the model is adapted, and the optimal insulin infusion is computed. To illustrate the performance of the personalized patient module, estimates on P1–P5 and the actual measurements on CGM from clinical trial is shown in Figure 13. One can see that personalized patient module provides more accurate forecasts of the BGL variations as indicated by low RMSE (Table 2).

**Note 2:** Adaptive patient model was validated against the data as well as conventional Type 2 models. As usual, T2D dynamics are nonlinear, and computing model parameters is quite cumbersome. However, the adaptive model presented in the paper mimics the nonlinear dynamics efficiently, and the results of the comparison are not provided in the paper due to space constraints.

#### 3.4.2. Personalized Insulin Management Module

The proposed personalized insulin management system uses the BGL measurements and patient model to compute the optimal insulin infusion to maintain BGL within recommended limits. To compute the optimal insulin infusion, it solves a multi-time step optimization model whose objective is to reduce the BGL excursion through proper choice of insulin, and the constraints for the problem are recommended BGL limits, patient dynamics, and limits on insulin infusion rates.

##### Perturbation Terms for Geriatric Factors and Nutrient Intake

Traditionally, an MPC works in three steps: (i) update measurements, (ii) compute control input using model, and (iii) apply the first among the computed control inputs in every iteration as with traditional MPC approaches. The rest of the computed control inputs are discarded by the MPC, and the procedure repeats during each time-step. This is called the receding horizon control. The LB-MPC is a new concept wherein the idea is to fuse factors obtained from domain informed data-based modeling tools (e.g., machine learning) to learn models or control actions (e.g., reinforcement learning).

More recently, LB-MPC is proliferating various application domains due to their ability to model even abstract concepts within model or control actions [56]. To personalize and precisely compute insulin infusion considering patient’s geriatric conditions, fusing domain informed data-based models with conventional mathematical models is required. To this extent, the LB-MPC approach is used as the personalization module in our HDT. The idea is to use parametric models wherein during each computation there are parameters from mathematical models and perturbation parameters that model patient specific aspects (e.g., insulin sensitivity or diet influences). In our analysis, the perturbation parameter $\delta_{a}$ represents the effect of geriatric influences on BGLs. Suppose that the LSTM forecast is denoted by $\hat{y}\left(k\right)$, whereas the model output is denoted by $\hat{G}\left(k\right)$. The LSTM models capture future behaviors based on past measurements, whereas the mathematical model is used to observe the insulin infusion effect in the future on BGLs.

At each time epoch *k* the model is updated and the future control moves are computed by solving a multi-time step optimization model. The LB-MPC uses the adaptive model of the patient and computes insulin infusion that manages BGL within specified limits. Therefore, by introducing the perturbation term, both past and future behaviors are included in the LB-MPC formulation. However, as the adaptive model is updated during each time epoch, and computation runs for the prediction horizon $N_{p}$, the model is not updated during computation, but rather after each time epoch. With an abuse of notation to denote the model parameters to be varying, we include the index *k*. The mathematical disturbance parameter during each time epoch is given by,

(11) $$\varepsilon_{1}\left(k\right)=G\left(k+1\right)-\zeta_{1}\left(k\right)(G\left(k\right)-\zeta_{2}\left(k\right)I\left(k\right)-\zeta_{3}\left(k\right)CHO\left(k\right)\;\forall \left\{k=1,2,\cdots ,N_{p}\right\},$$

Equation (11) models the static disturbance term at time instant k+1. However, the disturbances could vary depending on the intermittent and time-varying aspects. Therefore, to model the geriatric effects, a perturbation term is added to the model parameters:

(12) $$G\left(k+1\right)=\left(\zeta_{1}\left(t\right)+\delta_{a}\left(k\right)\right)G\left(k\right)+\zeta_{2}\left(t\right)I\left(k\right)+\zeta_{3}\left(k\right)CHO\left(k\right)+\varepsilon_{1}\left(k\right),\;\forall \left\{k=1,2,\cdots ,N_{p}\right\},$$

The perturbation term is computed as,

(13) $$\delta_{a}=\eta_{1}\times \frac{\hat{y}\left(k+1\right)}{G(k+1)}+\eta_{2}\times \frac{\hat{y}\left(k+1\right)-\hat{y}\left(k\right)}{h},\;\forall \left\{k=1,2,\cdots ,N_{p}\right\},$$

where $\eta_{1}$, $\eta_{2}$ and *h* denote the adaptation parameters and sampling time, respectively. The values of adaptation parameters $0<\eta_{1}<1,$$0<\eta_{2}<1$. The first term in the perturbation term (13) models the factor that adapts the perturbation parameter depending on the error between the model estimate and LSTM estimate. The second term models the adaptation w.r.t the slope of the BGL forecasts from the LSTM. These two terms together model the perturbation term that captures geriatric effects on the patient. In essence, the LSTM model provides predictions fusing food and insulin influences. These aspects are captured using the parameter.

The LB-MPC tries to compute the optimal insulin that would maintain the BGL within recommended bounds for each patient. Therefore, the decision variable is insulin infusion. The objective aims to minimize insulin costs, and a slack cost term and variable are added to allow for small constraint violations due to numerical issues that could be avoided. The constraints are the personalized patient model, recommended BGL bounds for the patient, insulin infusion, and non-negative slack variables. The LB-MPC solves the optimization problem during each time-horizon and the optimization model is,

(14) $$\begin{array}{cc} \; & \min_{I(k)}\sum_{k=1}^{k+N_{p}}C^{T}I\left(k\right)+C_{\gamma }\gamma \left(k\right)\\ \; & s.t.\\ \; & G\left(k+1\right)=\left(\zeta_{1}\left(k\right)+\delta_{a}\left(k\right)\right)G\left(k\right)+\zeta_{2}\left(k\right)I\left(k\right)\\ \; & \;\;\;\;\;\;\;\;\;\;\;\;\;+(\zeta_{3}\left(k\right)+\delta_{c}\left(k\right))CHO\left(k\right)+\varepsilon \left(k\right)\\ \; & G_{min}-\gamma \left(k\right)\leq G\left(k\right)\leq G_{max}+\gamma \left(k\right)\\ \; & I_{min}\leq I\left(k\right)\leq I_{max}\\ \; & \gamma \left(k\right)\geq 0\;\;\;\;\;\forall k:1\leq k\leq N_{p} \end{array}$$

where γ(k) is the slack variable, which introduces a soft constraint for modeling small variations in BGL over recommended limits.

Here, $C^{T}$ and $C_{\gamma }$ denote the cost for constraint violations, which is usually a very high value. The optimization model’s output is the insulin infusion for time instants $\left(k+1\right)\cdots \left(k+N_{p}\right)$. In the MPC, the first among the computed insulin dosages—i.e., k+1 is used as the insulin dosage while the rest of the computed ones are discarded—and the procedure is repeated during the next time-instant. This is called the receding horizon or moving horizon approach that is used within the MPC. The prediction horizon $N_{p}$ is the duration for which the control inputs are computed, and $T_{s}$ is the sampling period for the LB-MPC. In our approach, a sampling period of 15 min was selected, and a prediction horizon of 4 (one hour into the future) was used for most patients. The sampling period of 15 min was selected to capture the dynamics of the patient physiological model. The parameter variations model the patient-specific variations due to: food intake, insulin efficiency, comorbid conditions, and other aspects. By designing an MPC using these models and embedding knowledge of patients on the glycemic range, the proposed MPC becomes an LB-MPC. Due to this integration and modeling, the LB-MPC becomes E-T2D specific, i.e., it includes the geriatric influences inherently in the patient model. Further, by using contextual patient data on multiple drugs, the glycemic range is defined. Using these aspects the LB-MPC could not only integrate food influences on individuals but could also provide personalized optimal dosing to patients by considering their geriatric conditions. The software implementation aspects of the HDT are discussed in Appendix A. The detailed outcome of the LB-MPC managing E-T2D is provided in the results section.

## 4. Results

This section illustrates the HDT’s efficacy in managing diabetes using personalized recommendations, providing diagnostics, predictions, and other management. This is done in two steps:

(i) Clinical trials for 14 days on 15 elderly patients to collect patient-relevant data and blood glucose measurements. This is used for our modeling wherein an adaptive patient model, LSTM, STA, XAI, and other models are obtained. In this phase, infusions were done with insulin pumps pre-programmed based on diabetologist recommendations.
(ii) Simulations with the model to compute precision insulin infusion to avoid BGL excursions in E-T2D patients exploiting the different HDT models. The MPC presented is a simulation result that uses the patient model obtained from clinical trials. The diabetologist verified these results and confirmed the findings. Moreover, its implementation with an insulin pump is feasible through pre-programmed inputs from insulin pumps, as with clinical trials. However, due to constraints in volunteer recruitment and re-admissions, only simulation results are provided in the paper.

From clinical trials, data collected from 15 elderly diabetic patients is presented. The patients had co-morbid conditions and multiple drug intakes. The volunteers were recruited after initial clinical and ethical clearance. Then the data was collected for the initial study and is illustrated in the first section. This data had multiple facets: contextual data, medical records, patient-specific data, images, annotations, temporal data, and others. Similarly, time-series data was collected for blood glucose measurements. Alongside other data such as activity levels, nutrient intake, food timings, insulin infusion, etc., were used by HDT models to create knowledge from the aggregated data. The data was collected using the IoMT architecture proposed in our study.

Similarly, using dynamical models, time-evolution and optimal insulin infusion could be computed by using the LB-MPC approach described in this paper. During insulin computations, the CHO, and current blood glucose levels are the inputs. However, for geriatric patients, the control BGL rate could vary depending on their personal conditions. Currently, such aspects are identified from historical information and diabetologist recommendations. The DT in this paper uses information from diabetologists that are contextual patient data: hyper and hypo constraints that could be achieved for a particular patient. However, by fusing dynamical models with domain-informed data-based models, the HDT obtains deeper insights and proposes suitable actions considering aspects of geriatric conditions of the individual patients. This extends the current capabilities for personalizing and managing diabetes using conventional tools. In what follows the results from these aspects are presented.

### 4.1. Clinical Data Description

During the data collection phase, 15 elderly patients with varied geriatric conditions were recruited at Jothydev’s Diabetes Research Center (JDRC), Trivandrum, India. The data was collected from 2017–2018. The data collection had two phases: (i) patient recruitment and initial data collection and (ii) clinical data collection. In the patient recruitment phase, contextual data and medical data such as patient name, co-morbid conditions, multiple drug intake, patient-specific data, average calories, anthropometric data (e.g., body mass index), and others were recorded. These data were converted to contextual data as a JSON file (JavaScript Object Notation). Further, the data was entered and stored as medical records in the hospital.

**Note 3:** The patient’s name and other personal aspects were coded with suitable protocols to avoid leakage of personal data. The registered ethics committee registration number for this data collection process is ECR/115/Indt/KL/2013/RR-16 issued under rule 122DD of the Drugs and cosmetics rules 1945, Government of India.

**Figure 14:**
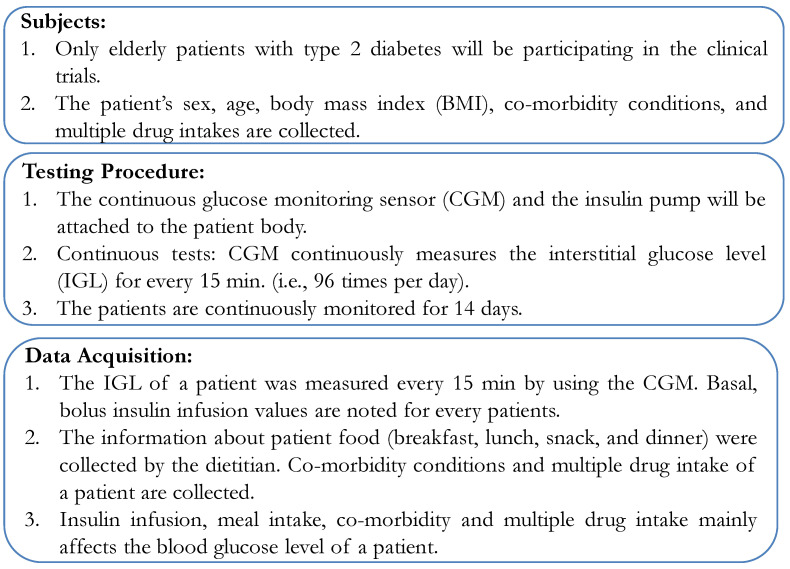
Testing Protocol.

Having recruited volunteers and collected contextual data, the next step was to formulate the clinical trials. As this involved patient data collection, a protocol needed to be established for the clinical trial. First, the protocol volunteers were defined. Only E-T2D patients were recruited for the clinical trials whose contextual data had already been obtained. Second, the testing procedure was to use the CGM for measuring continuously the BGL. In this condition, the patients would be continuously monitored in the hospital in a restrictive environment. Third, in the data acquisition phase, BGL was measured every 15 min with insulin infusion using an insulin pump. The basal and bolus values of insulin infusion were recorded from the pump. Additionally, information about the patient’s food intake: breakfast, lunch, snacks, and dinner was collected by a dietitian based on the food exchange list that was being served to the patients. The testing protocol used in our study is illustrated in Figure 14.

### 4.2. Clinical Data Collection

During the clinical data collection period, blood glucose levels, CHO intake, and insulin infusion for the patients were collected for 14 days. The freestyle Libre Pro CGM sensor was used to collect the glucose level. The sensor insertion, removal, and troubleshooting were performed by a trained physician at JDRC. The insulin was administered by using the Medtronic 722 insulin pump. The data was collected every 15 min and was transmitted to the cloud to be stored/retrieved for modeling. The CGM sensor had to be calibrated with finger stick blood glucose measurements 2 or 3 times per day. The patient details and contextual data are shown in Table 3.

**Figure 15:**
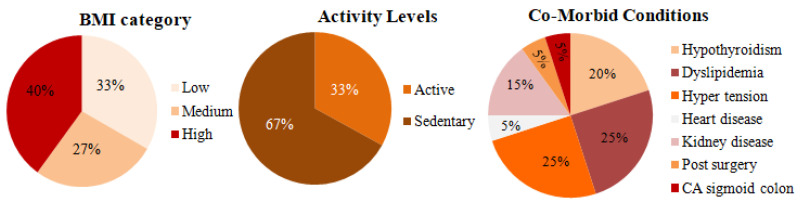
Patient data.

Figure 15 shows the pie chart representation of 15 E-T2D patients’ data collected at JDRC. The first figure shows the BMI distribution among the volunteers used in our study. Based on BMI the patients are classified into three categories: patients’ BMI values between 18–25 are considered low BMI, between 25–30 are categorized as medium BMI, and above 30 is considered as high BMI. The distribution is indicative of the actual occurrence of patients. As for activity levels, 33% of the people were active while the remaining 67% were either moderate or sedentary. A close look at the co-morbidity of the patients under study: the distribution shows Hypothyroidism, dyslipidemia, hypertension, heart disease, kidney disease, post-kidney and heart surgery, and sigmoid colon, and multiple drug intake.

**Figure 16:**
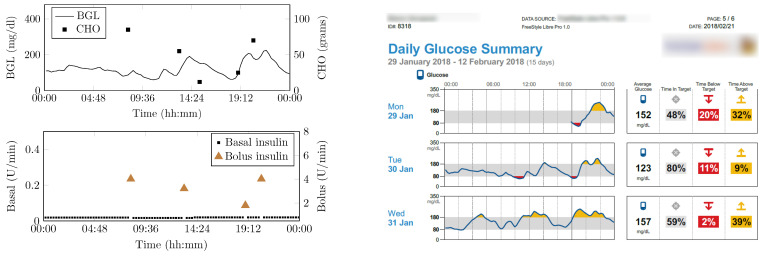
Snap-shot of one day data collected from a patient.

Figure 16 shows the snapshot of one-day data collected from patients. It contains BGL variations, insulin infusion and CHO present in patient food. The CHO present in the food was determined and logged by a trained dietitian at the time of the study. Three meals, and snacks timed to vary around 07:30 (Meal 1), 13:30 (Meal 2), 16 (snack), 20:00 (Meal 3) ± 30 min. The number of meals may vary based on the patient’s condition and was recorded as contextual data.

### 4.3. Vital Signs and Activity Data

**Figure 17:**
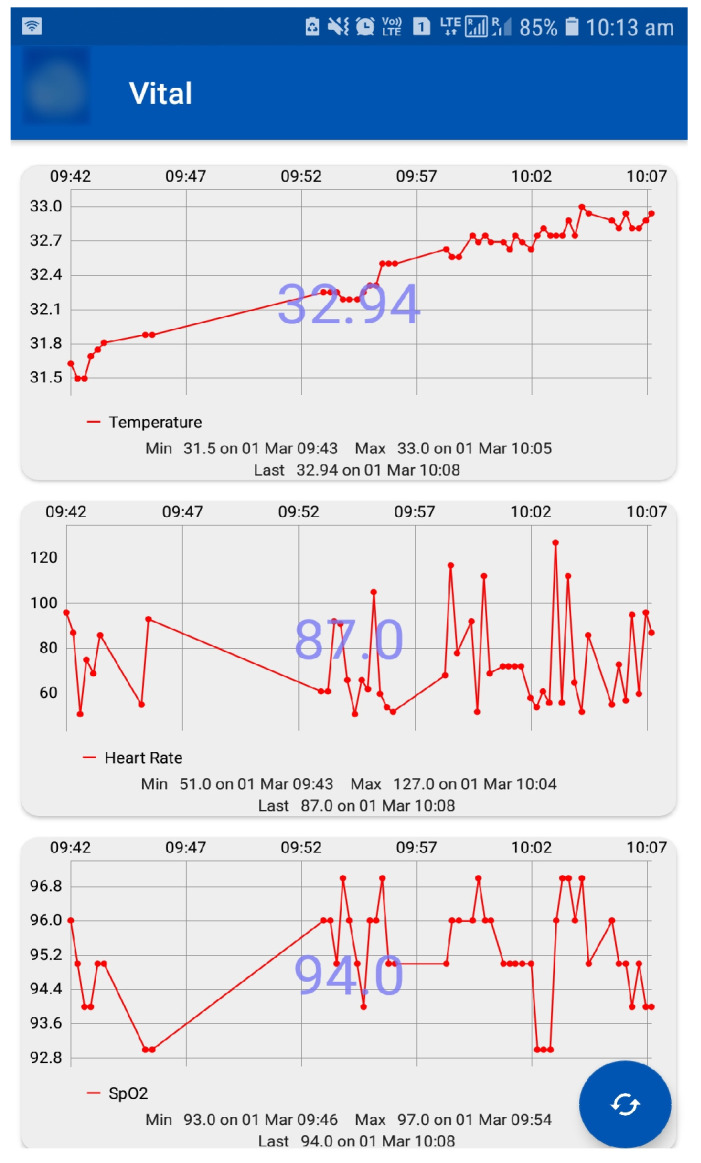
Vital Sign Monitoring Using Mobile App—Top figure shows the body temperature data, the middle shows the heart rate data, and the bottom figure shows SpO${}_{2}$ data.

As the BGL is influenced by the activity levels recording them helps identify their influences. Especially, activity influences patients having co-morbidity and multiple drug intake is very complex to understand. Therefore, by exploiting the activity data recorded through an integrated sensor that measures vital signs (e.g., body temperature) to activity levels using an accelerometer is used as described earlier. These values are recorded using suitable sensors and interfaced to a mobile app. The sensors and mobile app are an integral part of the IoMT architecture. The data displayed on the mobile app is shown in Figure 17.

In our analysis, activity levels were classified into active, moderate, and sedentary. The lifestyle of a patient was detected by using the CC2650 sensor tag that has an accelerometer to detect the patients activity. This data is transferred to the cloud via the edge node using Wi-Fi. Activities can be detected by x, y, and z axis positions. This activity information about the patient is helpful to personalize the insulin infusion.

### 4.4. Prediction Module Data Analysis

**Figure 18:**
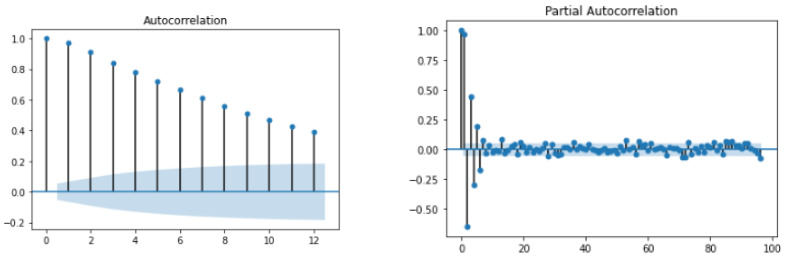
ACF and PACF plot for $P_{1}$.

During the exploratory data analysis (EDA), studies were performed on contextual and temporal data collected from clinical and pre-clinical trial phases, and it would be helpful to enumerate the data types for the contextual and temporal data used in this analysis (e.g., CHO, BGL, activity, etc). Then the data was fused to perform EDA for analysis. First, the auto-correlation function (ACF) and partial auto-correlation function (PACF) were evaluated to understand the dependency of the current samples on the past values. The ACF and PACF plots for patient $P_{1}$ are shown in Figure 18. An analysis of the ACF shows that there is a very strong correlation between the current value and past samples. This shows that regression-based models can estimate the current BGL from past samples for certain patients. However, the PACF plot shows that not more than two samples near to the current one may be useful in predicting it. Therefore, time-varying intermittent behaviors, causal variables, and exogenous factors may influence BGL predictions.

### 4.5. HDT Diagnostic Module Results

As stated earlier, the HDT uses two types of models: domain-informed data-based and dynamical models. This section first describes the results of domain-informed data-based models and how fusing data helps understand the influences of various geriatric and influencing factors. This section illustrates HDTs’ ability to detect interpersonal variations considering time-series data patterns. Patients’ temporal data is analyzed for motifs that denote the existence of patterns in time series that could discern scenarios with similar patterns, but the magnitudes may differ due to certain factors. By detecting motifs influencing factors could be found.

#### Motif Detection for Interpersonal Variations Detection

**Figure 19:**
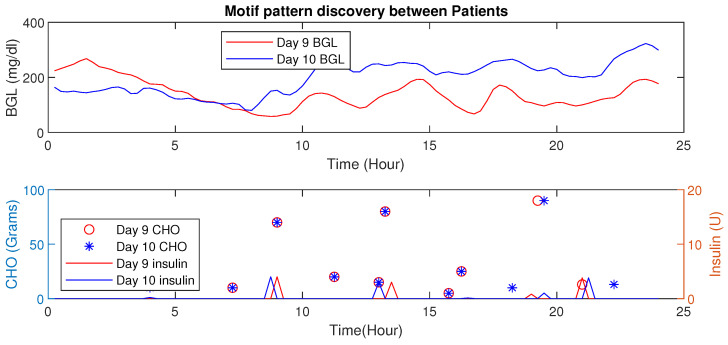
Time-Series Analysis: Motif Discovery between days 9 and 10 of P1.

This example illustrates the HDTs’ ability to detect changes in activity through data-driven techniques. As an example, time-series data for patient $P_{1}$ is presented wherein two different scenarios are shown for day 9 and day 10 (see, Figure 19), and the euclidean distance is 5.31 (mg/dL in minutes) shown in Table 4. These are two simultaneous days with similar patterns of diet intake and insulin infusion. Nevertheless, the patient $P_{1}$ had an exercise activity; walking for about 30 min on day 9 at morning 8:30 AM. Consequently, one can observe that the patterns for BGL are quite different or vary in magnitude. Day 9 with activity showed a lower BGL than day 10. This is primarily due to walking activity. Using such patterns the DT learns intricate aspects about the patient, such as sensitivity to activity or even a particular diet. This is a very important feature for personalization; as for managing BGL, the DT may suggest activities on a particular day whenever similar patterns are detected. Note though that, the result presented here is for illustrative purposes only. Similarly, patterns were observed for other aspects, such as multiple drug intake, nutrient variations, and others. The example demonstrates DTs’ ability to draw additional conclusions going beyond existing features with other techniques. Besides, such observation could be embedded in diabetes management as well.

### 4.6. Motif Based Intra-Personal Variations Detection

**Figure 20:**
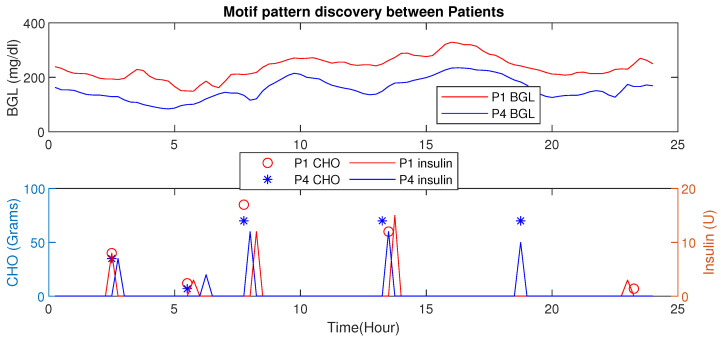
Motif Discovery between patient $P_{1}\&P_{4}$: Motif shows days with similar patterns. Patient $P_{4}$ has lower BGL than $P_{1}$ due to active lifestyle and relatively lower co-morbid conditions.

This section presents the HDT data-driven model’s capability to detect intra-personal variations and draw conclusions about personalization. In this analysis, patient $P_{1}$ has co-morbid conditions and multiple drug intake compared to patient $P_{4}$ with a liver ailment. However, their age, activity levels, and diet intake are similar. Figure 20 shows the blood glucose level motifs for the two patients with similar CHO intakes. Even with their initial conditions being similar, the pattern indicates that patient $P_{1}$ has a higher BGL compared to patient $P_{4}$ in this time period. This is mainly due to co-morbidity and multiple drug influences. Similarly, the patient $P_{4}$ maintains a better BGL profile than $P_{1}$.

Learning such aspects from data is not possible with the current models available in the literature.

**Figure 21:**
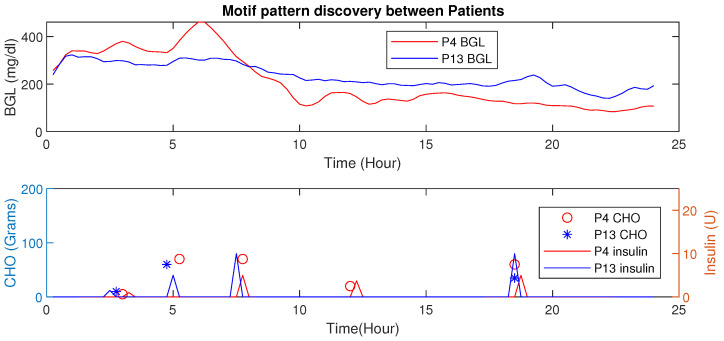
Time-Series Analysis: Motif Discovery between $P_{4}\&P_{13}$—$P_{4}$ has a lower magnitude of BGL profile even with high average CHO and less insulin infusion.

Similarly, comparison of the motif between two patients $P_{4}$ and $P_{13}$ is shown in Figure 21. The BGL magnitude for $P_{4}$ is less compared to $P_{13}$ this is because of activity levels, co-morbidity, and multiple drug intake. Even though the average CHO per day for $P_{4}$ is high, the patient has a lower magnitude of BGL.

These results not only illustrate the HDTs’ ability to use intra-personal variations to personalize diabetes management but also prescribe the infusing of insulin depending on their conditions. By fusing data with patient models in an HDT, such capabilities could be enabled, such aspects are unexplored for conventional models.

### 4.7. Personalization Module with XAI

**Figure 22:**
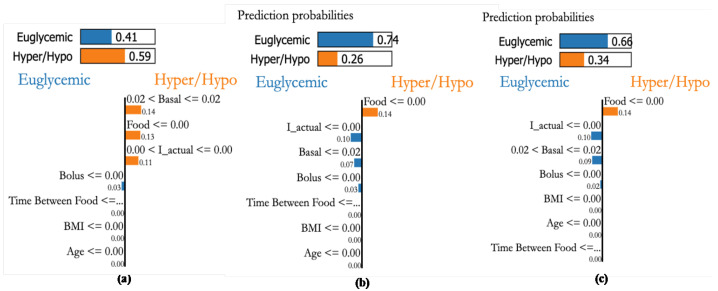
Explanations for three randomly selected samples of patient P1. (**a**): An actual sample in the hyperglycemic range detected with high probability through XAI as basal value of 0.02, (**b**): sample 2 that is in Euglycemic range being detected with high probability with Insulin actual values, (**c**): Sample in Euglycemic range detected with probability of 0.64 through basal and bolus.

Personalized explanations based on recorded samples help avert critical conditions. The explanations provided for 3 different samples are shown in Figure 22a–c. The bars on the top provide the probability of the sample to within the euglycemic range or otherwise. Figure 22a shows a case with a higher probability (0.59) to be in the hypo/hyper range due to basal, food, and effective insulin. Whereas the second sample indicates food as a major factor (see, Figure 22b). Similarly, sample Figure 22c shows that food is a major factor pushing towards hyper-probability. This indicates that the patient is influenced by CHO intake and her bolus insulin has to be increased.

**Figure 23:**
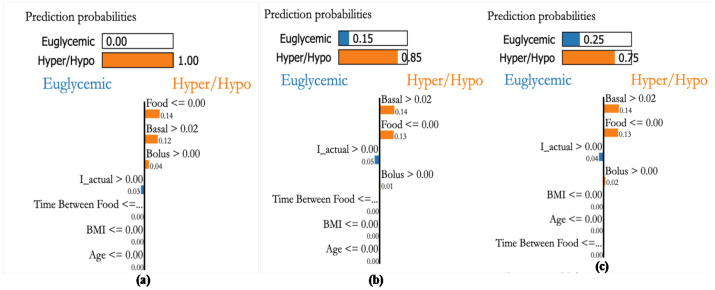
Explanations for three randomly selected samples of patient P1. (**a**): Sample showing hyper condition and explained through basal and bolus values even though CHO is low, (**b**): A hyper glycemic sample explained through basal values even when the food CHO is low, and (**c**): random sample detected to be hyper glycemic based on basal and bolus values.

Patient P3, a female aged 36 and having a kidney transplant, has an average daily CHO consumption of 300 g. A glycemic range of 80–140 [mg/dL] is recommended. Figure 23a shows a case with a higher probability of being in hypo or hyper conditions because of food, basal, and bolus insulin values. The second sample Figure 23b also indicates the higher probability of hypo/hyper conditions, the third sample shown in Figure 23c also indicates that higher probability in hypo/hyper conditions. From this, we can see that the personalization module with XAI reveals that both basal and bolus (effective insulin) are the main factors influencing the BGL of the patient as in Figure 23 suggesting a sensitive insulin infusion due to co-morbid conditions.

### 4.8. BGL Management through Precision Insulin Infusion

This section describes the HDT-based BGL management module for E-T2D patients that handles their geriatric challenges. The module personalizes insulin infusion to manage BGL within the target range. To compute the insulin infusion, the HDT has a patient model with parameters modeling their food and insulin influences on BGL. The current BGL measurements are obtained from sensors; food influences are obtained from the mobile app and food nutrient prediction algorithm. The HDT-based BGL management module computes the precise insulin infusion for the patient considering geriatric conditions. The personalized optimal insulin dosage to maintain the BGL within limits is computed using the MPC approach proposed in this paper.

**Figure 24:**
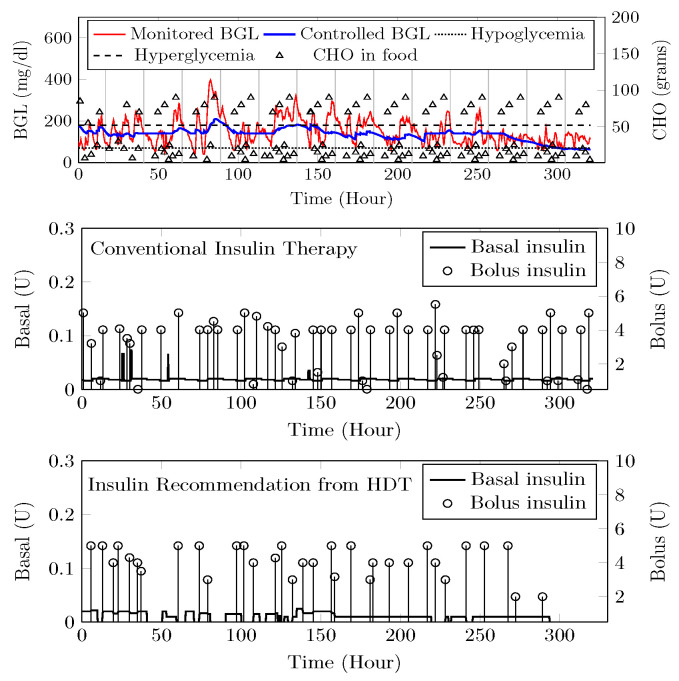
Insulin recommendation and BGL for $P_{1}$—Top panel shows the comparison between uncontrolled BGL variations during the clinical trial and controlled BGL using HDT framework. The middle plot shows insulin infusion during the clinical trial, whereas insulin recommendation from HDT is shown in the bottom plot.

The HDT will mimic the patient’s glucose dynamics, and the MPC will compute the BGL within the target range. The results were studied through simulations on 15 patient models for 14 days of data on food and insulin infusion. The results are validated against the clinical data. A control band of 80–180 mg/dL^−1^ is recommended for Patient $P_{1}$ by the physician, mainly due to co-morbid conditions and patient history. Figure 24 shows the comparison between uncontrolled clinical trials and HDT-based control scenarios. During clinical trial, $P_{1}$ had 363 hyper and 113 hypo events, whereas this was reduced to 99 hyper and 72 hypo events with LB-MPC.

**Figure 25:**
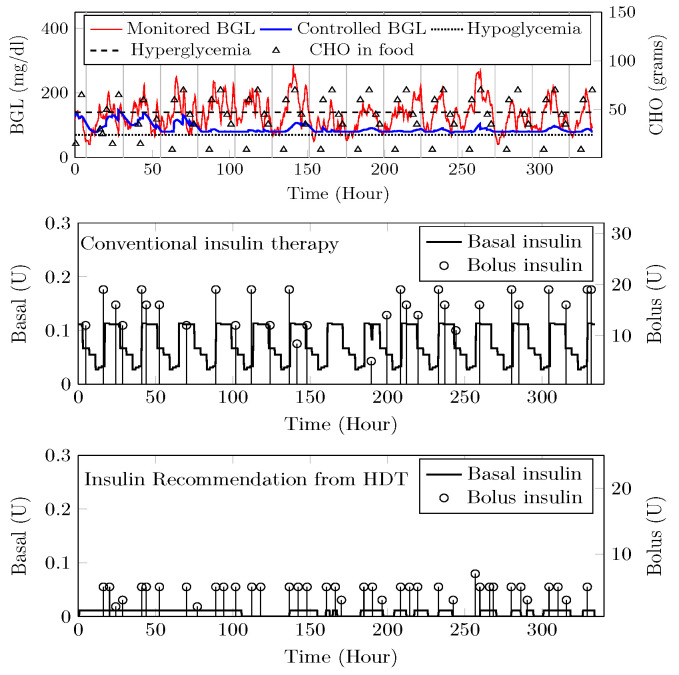
Insulin recommendation and BGL for $P_{2}$—Comparison between uncontrolled clinical trial and controlled BGL excursions with HDT framework is shown in the top plot. The middle plot shows the insulin recommendation during the clinical trial, and the bottom plot shows the insulin recommendation from HDT.

A target range of 80–150 mg/dL was recommended for Patient ($P_{2}$) by the physician based on patient. Comparisons between clinical trials and personalized APs’ BGL regulation are shown in Figure 25. Patient $P_{2}$’s clinical data were collected for 13 days because of the malfunction present in the CGM device. Insulin recommendation from HDT maintains the BGL within the bands specified by the physician even with high CHO consumption, co-morbid conditions multiple drug intake. During clinical trials, $P_{2}$ is subjected to 478 hyper-glycemic and 104 hypoglycemic events. whereas this was reduced to 28 hyper events, and there are no hypo events with insulin recommendation from HDT. Furthermore, the patient used 350 Units of insulin in 13 days, but with LB-MPC, this is reduced to 250 Units, i.e., a 28.5% reduction over 13 days, the number of insulin infusion events was reduced from 96 to 70 times per day. This result demonstrates personalized AP’s ability to regulate BGL in patients with high CHO intake, co-morbidity, and multiple drug intake.

**Figure 26:**
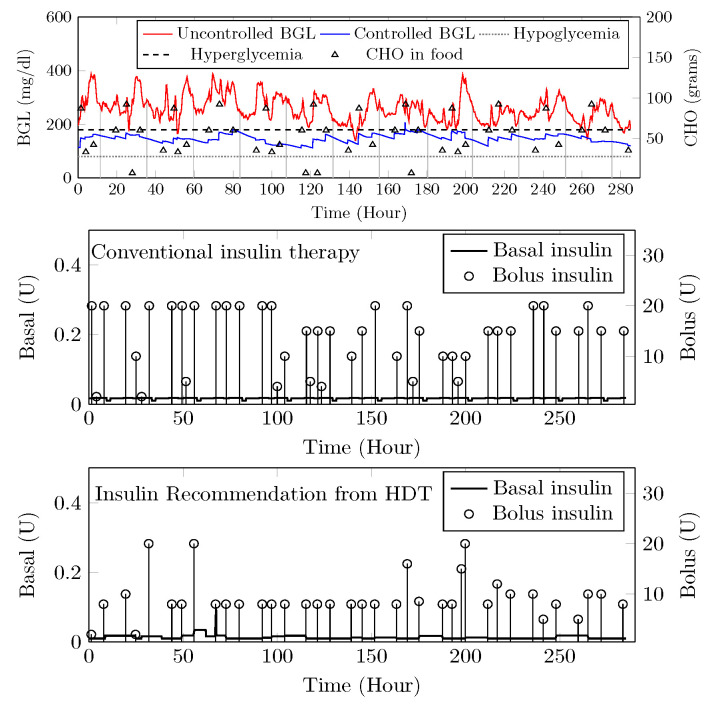
Insulin recommendation and BGL for $P_{13}$—Comparison between uncontrolled clinical trial and controlled BGL excursions with HDT framework is shown in the top plot. The middle plot shows the insulin recommendation during the clinical trial. The bottom plot shows insulin recommendations from HDT.

Patient ($P_{13}$) suffers from hyperglycemia during clinical trials, even with insulin infusion. Moreover, $P_{13}$ had heart surgery and hypertension. The target BGL range recommended by the physician is 90 to 180 mg/dL. Comparisons with clinical trials and personalized AP control are shown in Figure 26. While in uncontrolled trials, there were 1114 hyper-glycemic episodes, there were no events recorded with LB-MPC. Furthermore, patient ($P_{13}$) was administered 550 units during the trial period, whereas 485 units were required for maintaining the BGL using the HDT, reduced by 14.5%, the insulin infusion events were reduced from 96 to 80 times per day. These results demonstrate that the insulin recommendation from the HDT can not only regulate the BGL for patients with significant hyper-glycemic events but with reduced insulin infusion.

The time-in-range, time spent in hyper, and time spent in hypo conditions comparison between conventional insulin therapy and HDT-based personalized insulin recommendation is shown in Table 5.

**Figure 27:**
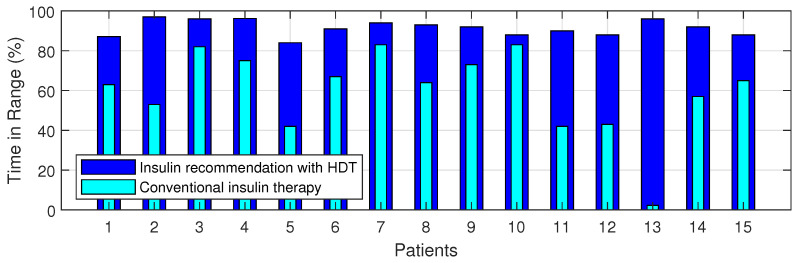
Percent improvement in Time in Range for 15 patients.

**Figure 28:**
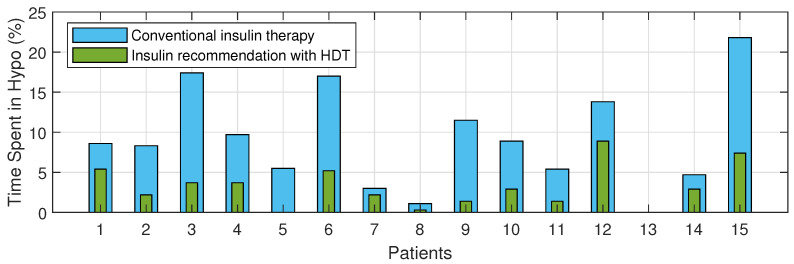
Percent improvement in hypo events for 15 patients with HDT.

**Figure 29:**
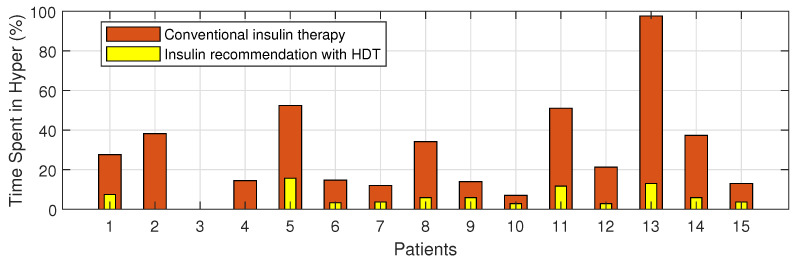
Percent improvement in hyper events for 15 patients with HDT.

The Percentage of improvement in TIR for 15 patients with HDT-based personalized BGL management is shown in Figure 27. This shows that the TIR is increased from 3–75% to 86–97%. Figure 28 and Figure 29 show the percentage of improvement in hypo and hyper conditions with HDT-based personalized BGL management. This shows that HDT-based personalized BGL management can reduce the time spent in hypo from 0–22% to 0–9%, and percentage of time spent hyper is also reduced from 0–98 % to 0–12%.

## 5. Conclusions

This paper presented a Human Digital Twin (HDT) framework for Elderly Type-2 Diabetes (E-T2D). The HDT enables personalization and precise insulin infusion considering specific patient aspects. Further, the HDT provided deeper insights by aggregating various data: contextual, clinical, patient-specific, etc. Second, the HDT has a suite of models that can leverage this data and provide outcomes that can be used to obtain deeper insights into E-T2D. These models combined deep-learning models for time-series forecasting, image processing, and others. Finally, a mathematical model that can adapt its parameter based on clinical data is also proposed. Exploiting the deep-learning tools and mathematical models, a learning-based MPC is proposed that can personalize insulin infusion depending on the patient’s geriatric conditions. The HDT design and development aspects are also discussed. Finally, the HDT implementation is deployed and tested on 15 E-T2D patients through clinical trials and simulations. Our results suggest that by personalizing diabetes treatment through HDT enhances the time-in-range, reduces the hyper and hypo events, and more importantly, reduces insulin infusion showing its efficacy in managing diabetes. Extending the clinical trials for more co-morbid patients and studying it for a large number of population is the future course of this study. 

## Figures and Tables

**Table 1:** LSTM results for P1–P5.

S. No	Prediction Error (%)	Normalized RMSE	Normalized MAE
P1	3.06	0.69	0.49
P2	5.12	0.96	0.89
P3	4.51	0.79	0.64
P4	5.02	0.92	0.85
P5	4.92	0.89	0.79

**Table 2:** Patient simulator Prediction results.

S. No	Prediction Error Range (mg/dL)		Percentage of Error (%)		RMSE
	Min	Max	Min	Max	
P1	−14.55	8.21	3.85	6.53	5.01
P2	−7.22	5.35	4.5	5.2	3.27
P3	−12.25	8.25	4.3	6.34	4.87
P4	−3.25	5.25	2.5	3.9	1.89
P5	−4.58	10.56	1.8	3.5	3.32

**Table 3:** Patient Details.

S. No	Age	Sex	BMI	Co-Morbidity Conditions	Target BGL Range (mg/dL)	Avg. CHO/day (g)	Life Style	Oral Drugs
P1	63	M	24.2	Hypothyroidism, Dyslipidemia	80–180	240	Sedentary	Glycomet 250 mg
P2	60	F	23.6	Hypothyroidism, Dyslipidemia, hypertension (HTN)	80–150	300	Sedentary	Metadoze-1, Hepsodil-1, Victoza-1, Blisto-1/2
P3	36	F	32.4	Dyslipidemia, HTN, kidney transplant	80–140	300	Sedentary	Trajenta Duo 2.5/500, Metadose IPR
P4	59	M	25.7	Liver problem	80–180	320	Active	Glycomet, Metadoze
P5	63	M	27.5	Dyslipidemia, Heart disease, CKD	80–180	240	Sedentary	Glycomet, Glimepiride, Aplazar, D-Rise, Metadoze, Carfer, Ril 2.5, Preganerve, Roliptin, Trajenta, Cilnipres
P6	72	M	25.1	HTN, mild NPDR	80–180	280	Active	Kombiglyza 5/500, Jardiance-25 mg, Cetapin-500
P7	79	M	32.1	CA sigmoid colon, CKD, HTN	80–150	220	Active	Glycomet 300 mg, Metadoze.
P8	80	F	31.9	CKD, Hypertension	80–180	20	Sedentary	GP-0.5, Metadoze
P9	58	F	23.1	Hypothyroidism, Dyslipidemia	70–180	300	Sedentary	Metadoze-1, Roliptin
P10	55	M	23.2	Dyslipidemia, CKD	80–180	234	Sedentary	Metafort 500 mg, victoza injection
P11	57	M	30.1	Hypothyroidism, Dyslipidemia	80–180	270	Active	Diafer 250, Jerdiance 100 mg
P12	66	F	30.8	Hypothyroidism, Dyslipidemia	80–140	300	Active	Metafort 1000 mg, Gride 1 mg, Victoza injection
P13	70	F	24.7	HTN, Coronary artery bypass graft surgery (CABG)	90–180	280	Sedentary	T-Semi-Amaryl 0.5, PPG Met 0.2
P14	65	F	34.5	Dyslipidemia, HTN, Anemia, CABG	80–140	320	Sedentary	Diafer 250
P15	62	F	29.1	CKD, HTN	90–180	250	Sedentary	T-semi-Armyl 0.5 mg

**Table 4:** Motif Pattern Analysis—Intra patient analysis of P1.

S. No	Days Compared	Euclidean Distance
1	Day 1 & 2	3.88
2	Day 3 & 4	6.15
3	Day 5 & 6	5.55
4	Day 7 & 8	2.01
5	Day 9 & 10	5.31
6	Day 11 & 12	6.27

**Table 5:** Performance metrics for personalized AP.

S. No	HDT Based PM			Conventional Insulin Therapy		
	TIR (%)	Time Spent in Hypo (%)	Time Spent in Hyper (%)	TIR (%)	Time Spent in Hypo (%)	Time Spent in Hyper (%)
P1	87.1	5.4	7.5	63	8.6	27.6
P2	97	0	2.2	53	8.32	38.2
P3	96	3.7	0	82	17.4	0
P4	96.2	3.7	0	75	9.7	14.5
P5	84	0	15.7	42	5.5	52.4
P6	91	5.2	3.3	67	17	14.7
P7	94	2.2	3.7	83	3	12
P8	93	0.3	5.9	64	1.1	34.2
P9	92	1.4	5.9	73	11.5	14
P10	88	2.9	2.9	83	8.9	7
P11	90	1.4	11.7	42	5.4	51
P12	88	8.9	2.9	43	13.8	21.3
P13	87	0	13	2.3	0	97.6
P14	92	2.9	5.9	57	4.7	37.3
P15	88	7.4	3.7	65	21.8	13

## Data Availability

The original contributions presented in the study are included in the article. Further inquiries can be directed to the corresponding author.

## Appendix A

### Software Implementation

The software implementation aspects of the HDT are discussed in this section. The software architecture has two parts: front-end and back-end. The front-end was implemented with hypertext modeling language (HTML) pages orchestrated by Flask package from Python. Conditional statements and other aspects were implemented using Jinja2 templating engine for use with Python. The front-end and back-end were orchestrated using Python script. Data was stored in comma separated variable file and displayed using the graphical display tools in Python.

The description module has interfaces to collect the data and store it in the cloud using the IoMT architecture. The implementation was done on a private cloud using Python scripts. It collects the sensed information and stores the data for retrieval. The Python was used to create these databases and display the data. Similarly, the prediction module is based on time-series modeling, and packages in Python such as Keras, Stumpy, Numpy, and other standard packages are used for analyzing the time-series data. LIME Python package was used to implement the explainable AI (XAI). LB-MPC implementation was done with GLPK linprog in a Python environment to personalize the insulin infusion.

In the prediction module Python package, Keras was used to multi-variate and multi-time step prediction of BGL samples. Figure 7 shows the flow of BGL prediction using LSTM from Keras. The structured time series analysis was performed by using the TensorFlow package from the Python environment. This provides time-series trends to capture the hyper and hypo conditions. Similarly, the Python environment was used in the prediction module to analyze the food and time-series evolution.

To interpret patterns and their outcomes in the diagnostic module, the Python package Stumpy was used to analyze the motif patterns. The results were used to analyze the inter and intra-patient variabilities. By analyzing activity inferences, pattern recognition, and contextual mapping this module can give alerts to the patient.

To personalize the insulin infusion, the management module uses the LB-MPC. It was implemented using GLPK linprog from a Python environment to compute the personalized insulin infusion. The performance of the HDT-based personalized management was analyzed using various metrics (e.g., Time In Range (TIR), hypo, and hyper events). Software implementation was simplified with Python used across the stack for implementation and the choice was dictated by the hardware’s ability to work with the software selected as well.
